# Exposure to traffic-related air pollution during physical activity and acute changes in blood pressure, autonomic and micro-vascular function in women: a cross-over study

**DOI:** 10.1186/s12989-014-0070-4

**Published:** 2014-12-09

**Authors:** Scott Weichenthal, Marianne Hatzopoulou, Mark S Goldberg

**Affiliations:** Air Health Science Division, Health Canada, 269 Laurier Avenue West, K1A 0K9 Ottawa, ON Canada; Department of Civil Engineering, McGill University, Macdonald Engineering Building, 817 Sherbrooke Street West, H3A 0C3 Montreal, Quebec Canada; Division of Clinical Epidemiology, McGill University Health Center, 687 Pine Avenue West, H3A 1A1 Montreal, Quebec Canada

**Keywords:** Epidemiology, Heart rate variability, Blood pressure, Endothelial function, Traffic-Related air pollution

## Abstract

**Background:**

Traffic-related air pollution may contribute to cardiovascular morbidity. In urban areas, exposures during physical activity are of interest owing to increased breathing rates and close proximity to vehicle emissions.

**Methods:**

We conducted a cross-over study among 53 healthy non-smoking women in Montreal, Canada during the summer of 2013. Women were exposed to traffic pollutants for 2-hours on three separate occasions during cycling on high and low-traffic routes as well as indoors. Personal air pollution exposures (PM_2.5_, ultrafine particles (UFP), black carbon, NO_2_, and O_3_) were evaluated along each route and linear mixed-effects models with random subject intercepts were used to estimate the impact of air pollutants on acute changes in blood pressure, heart rate variability, and micro-vascular function in the hours immediately following exposure. Single and multi-pollutant models were examined and potential effect modification by mean regional air pollution concentrations (PM_2.5_, NO_2_, and O_3_) was explored for the 24-hour and 5-day periods preceding exposure.

**Results:**

In total, 143 exposure routes were completed. Each interquartile increase (10,850/cm^3^) in UFP exposure was associated with a 4.91% (95% CI: -9.31, -0.512) decrease in reactive hyperemia index (a measure of micro-vascular function) and each 24 ppb increase in O_3_ exposure corresponded to a 2.49% (95% CI: 0.141, 4.84) increase in systolic blood pressure and a 3.26% (95% CI: 0.0117, 6.51) increase in diastolic blood pressure 3-hours after exposure. Personal exposure to PM_2.5_ was associated with decreases in HRV measures reflecting parasympathetic modulation of the heart and regional PM_2.5_ concentrations modified these relationships (p < 0.05). In particular, stronger inverse associations were observed when regional PM_2.5_ was higher on the days prior to the study period. Regional PM_2.5_ also modified the impact of personal O_3_ on the standard deviation of normal to normal intervals (SDNN) (p < 0.05): a significant inverse relationship was observed when regional PM_2.5_ was low prior to study periods and a significant positive relationship was observed when regional PM_2.5_ was high.

**Conclusion:**

Exposure to traffic pollution may contribute to acute changes in blood pressure, autonomic and micro-vascular function in women. Regional air pollution concentrations may modify the impact of these exposures on autonomic function.

**Electronic supplementary material:**

The online version of this article (doi:10.1186/s12989-014-0070-4) contains supplementary material, which is available to authorized users.

## Background

Traffic-related air pollution is known to contribute to cardiovascular morbidity including both acute and chronic health effects [1-5]. While the precise biological mechanisms underlying these associations have yet to be fully elucidated, existing evidence suggests a range of biological pathways including increases in plasma viscosity, altered autonomic function, arrhythmia, impaired vasomotor function, and the promotion of atherosclerosis [4-6]. Moreover, as municipalities move towards the promotion of active transportation in urban environments, it is increasingly important to understand the potential health impacts of physical activity in close proximity to traffic emissions. Indeed, time spent in traffic and personal exposures to soot have each been associated with acute myocardial infarction (MI) [7,8], with a particularly strong association observed among women [9]. In addition, others have reported an association between ultrafine particle (<0.1μm) (UFP) exposures and ST-segment depression with a stronger association observed among women relative to men [10]. Furthermore, recent evidence suggests that women moving from low-traffic areas to high-traffic areas have an increased risk of MI [11]. However, a review of the long-term health effects of air pollution did not observe strong evidence for gender differences in air pollution health effects [3].

In the present study, we examined the impact of traffic-related air pollution on acute changes in physiological measures of cardiovascular function among women in Montreal, Canada. Specifically, we examined acute changes in blood pressure, micro-vascular function, and heart rate variability as these measures may be involved in underlying pathophysiological pathways linking air pollution to cardiovascular morbidity/mortality. Women were selected as the target population as existing evidence related to the acute cardiovascular health effects of some traffic pollutants such as UFPs largely reflects responses among men [4].

## Results

**Figure 1:**
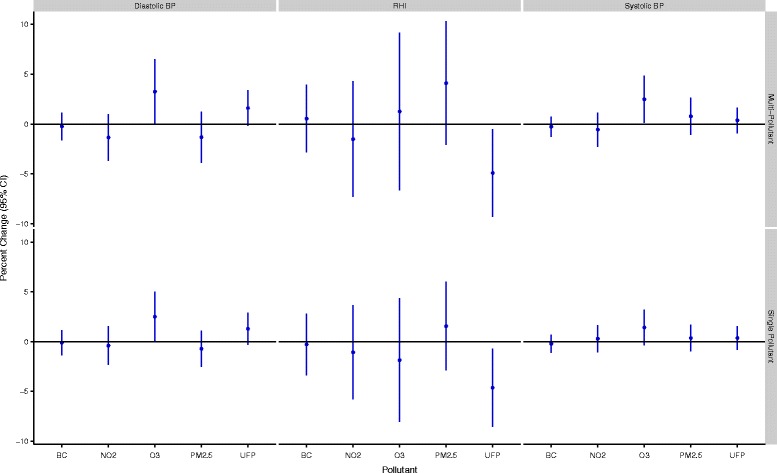
**Air pollution exposure and changes in RHI and Blood Pressure 3-hours after exposure.** All models are adjusted for ambient temperature during exercise, mean heart rate during exercise, and alcohol/caffeine consumption in the past 24-hours. Regression coefficients reflect interquartile range increases in exposure. Multi-pollutant models are mutually adjusted for all other air pollutants.

**Figure 2:**
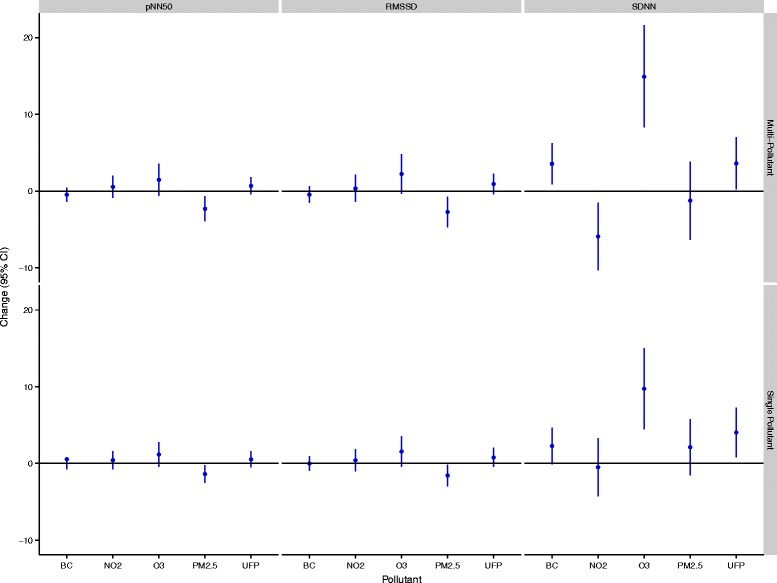
**Air pollution exposure and changes in time-domain measures of HRV over the 3-hour follow-up period.** All models are adjusted for ambient temperature during exercise, mean heart rate during exercise, and alcohol/caffeine consumption in the past 24-hours. Regression coefficients reflect interquartile range increases in exposure. Multi-pollutant models are mutually adjusted for all other air pollutants.

**Figure 3:**
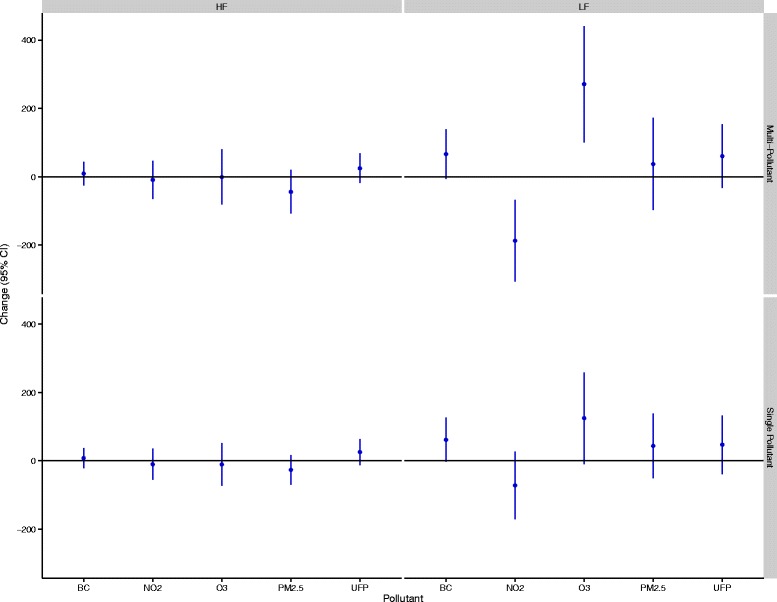
**Air pollution exposure and changes in frequency-domain measures of HRV over the 3-hour follow-up period.** All models are adjusted for ambient temperature during exercise, mean heart rate during exercise, and alcohol/caffeine consumption in the past 24-hours. Regression coefficients reflect interquartile range increases in exposure. Multi-pollutant models are mutually adjusted for all other air pollutants.

**Figure 4:**
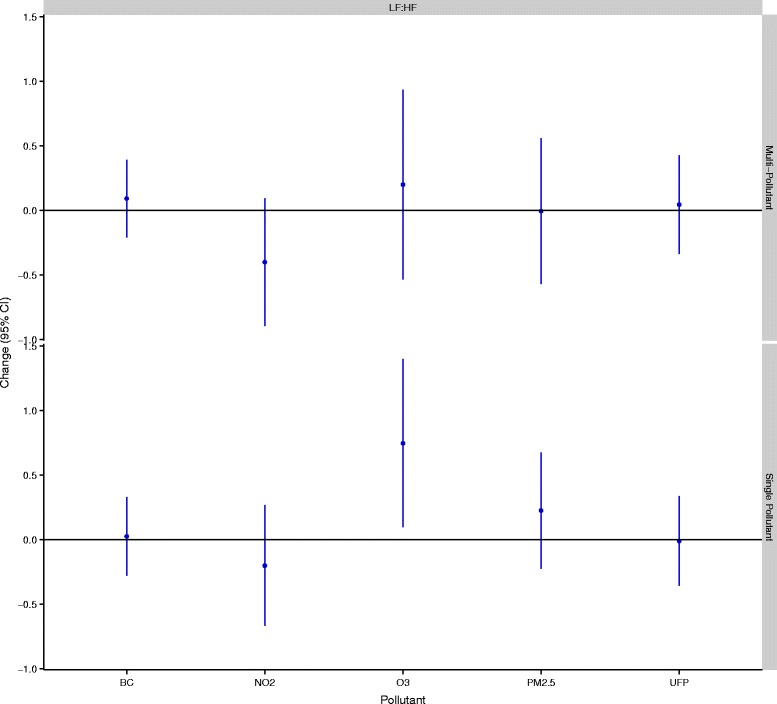
**Air pollution exposure and changes in the ratio of LF to HF.** All models are adjusted for ambient temperature during exercise, mean heart rate during exercise, and alcohol/caffeine consumption in the past 24-hours. Regression coefficients reflect interquartile range increases in exposure. Multi-pollutant models are mutually adjusted for all other air pollutants.

**Figure 5:**
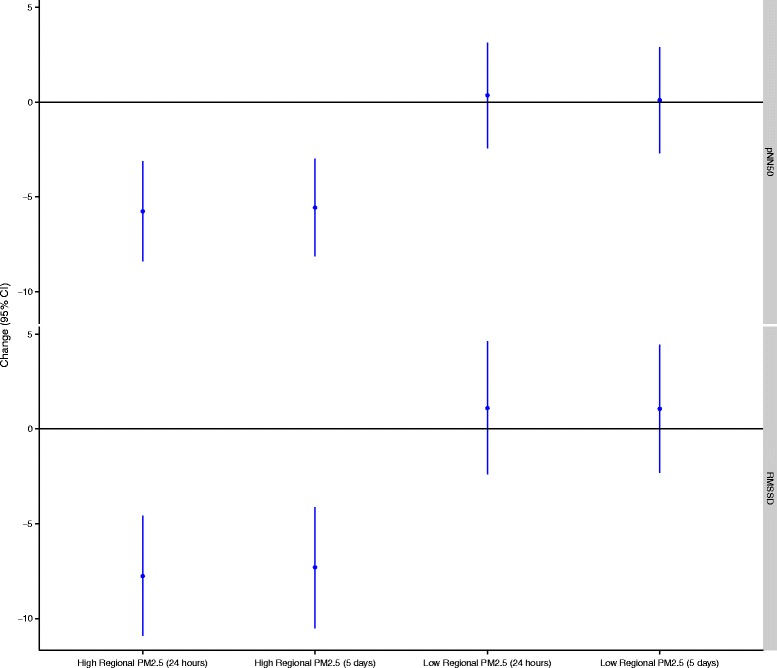
**Effect modification by 24-hour and 5-day mean regional PM**_**2.5**_ **in the relationship between personal PM**_**2.5**_ **and RMSSD and pNN50.** All models are adjusted for ambient temperature during exercise, mean heart rate during exercise, and alcohol/caffeine consumption in the past 24-hours. Regression coefficients reflect interquartile range increases in exposure. All models are mutually adjusted for all other air pollutants. The high/low cut points were 11.5 μg/m^3^ for 24-hour PM_2.5_ and 13.5 μg/m^3^ for 5-day PM_2.5_.

**Figure 6:**
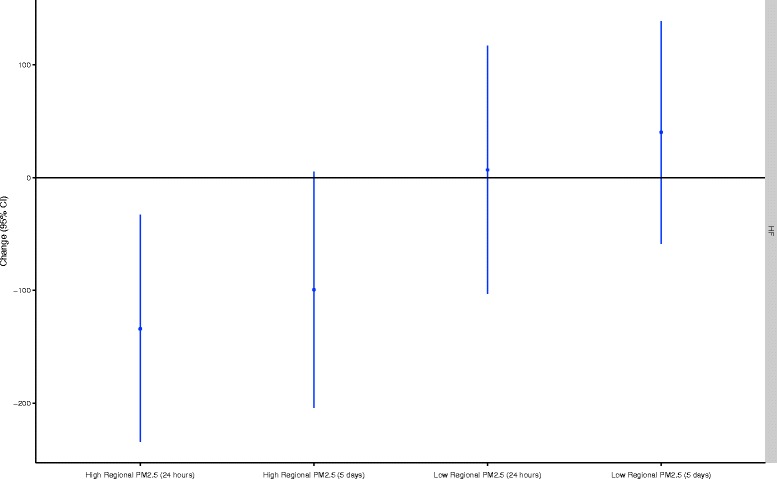
**Effect modification by 24-hour and 5-day mean regional PM**_**2.5**_ **in the relationship between personal PM**_**2.5**_ **and HF.** All models are adjusted for ambient temperature during exercise, mean heart rate during exercise, and alcohol/caffeine consumption in the past 24-hours. Regression coefficients reflect interquartile range increases in exposure. All models are mutually adjusted for all other air pollutants. The high/low cut points were 11.5 μg/m^3^ for 24-hour PM_2.5_ and 13.5 μg/m^3^ for 5-day PM_2.5_.

**Figure 7:**
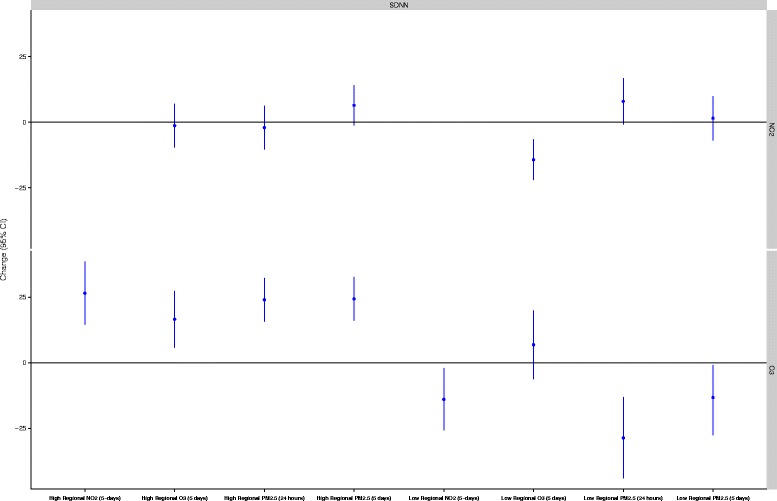
**Effect modification by regional air pollution in the relationship between personal NO**_**2**_ **and O**_**3**_ **and SDNN.** All models are adjusted for ambient temperature during exercise, mean heart rate during exercise, and alcohol/caffeine consumption in the past 24-hours. Regression coefficients reflect interquartile range increases in exposure. All models are mutually adjusted for all other air pollutants. The high/low cut points were 11.5 μg/m^3^ for 24-hour PM_2.5_, 13.5 μg/m^3^ for 5-day PM_2.5_, 6.59 ppb for 5-day NO_2_, and 26.47 ppb for 5 day O_3_.

In total, 53 women completed 143 exposure scenarios including 50 high-traffic routes, 48 low-traffic routes, and 45 indoor days. Air pollution and cardiovascular health data were available for nearly all of these routes although a small number of samples were lost because of instrument failure or technician error. In addition, a small number of data points were removed from analyses owing to changes much greater than for the study population as a whole. Specifically, 2 RHI measurements (out of 135) and 11 HRV measurements (out of 2791) were removed from analyses. The 2 RHI values were each collected from different participants whereas the HRV values reflected 5 separate subjects with 4 of the 11 points coming from a single subject who experienced large changes in the ratio of LF to HF on a single study day. Women ranged in age from 18 to 44 years (mean: 25 years), were predominantly Caucasian, and were generally not obese (Table 1). A small number of women reported regular use of medications including birth control (n = 9), irons pills (n = 1), and estrogen (n = 1). Descriptive data for baseline cardiovascular health measures are presented in Table 2. HRV data were similar to values observed in our previous study of healthy adults [12] and resting blood pressure levels were predominantly in the normal range with a small number of participants having high or low blood pressure values. The mean baseline RHI value was below 1.67, with 16 participants having values below 1.67 on all three study days. Reasons for low RHI values were not readily apparent; however, participants with higher diastolic blood pressures tended to have lower RHI values with each 10 mm Hg increase in diastolic blood pressure corresponding to a 0.106 (95% CI: -0.181,-0.00304 ) decrease in RHI (See Additional file 1: Figure S1). Adjusting for caffeine/alcohol consumption, age, race, BMI, recent illness, or second hand smoke exposure in the past 24-hours did not change this relationship. Personal exposures to air pollution varied substantially across study days (Table 3). Overall, spearman’s correlations between pollutants were low (r ≤ 0.33) but were similar to values reported previously for Montreal [13]. Within-route correlations were slightly higher; the strongest correlations were between UFPs and black carbon on the high route (r = 0.43), black carbon and NO_2_ on the low route (r = 0.54), and NO_2_ and O_3_ indoors (r = 0.55). Reasons for the low concentrations between pollutants may include several factors. For example, PM_2.5_ is primarily a regional air pollutant and thus in order to be strongly correlated with pollutants from local sources (e.g. UFPs and black carbon) high personal exposure routes would need to coincide with days with high regional PM_2.5_. Since routes were assigned randomly, this was not likely to occur. In addition, while diesel vehicles are a strong source of both UFPs and black carbon, gasoline vehicles are more prevalent in Canada and thus on a given route it is possible to experience high exposure to UFPs (from a large volume of gasoline vehicles) but low exposure to black carbon if large diesel vehicles are not encountered. In general, differences between routes were greatest for UFPs and black carbon. Specifically, UFP exposures were 2494 cm^−3^ (95%CI: -1589, 6578,) and 11,434 cm^−3^ (95% CI: 7928, 14,941) greater on the high-traffic route relative to the low-traffic and indoor locations, respectively. Black carbon exposures were 1012 ng/m^3^ (95% CI: 449, 1574) and 652 ng/m^3^ (95% CI: 34, 1270) greater on the high-traffic route relative to the low-traffic and indoor locations, respectively. Mean indoor levels of some air pollutants were similar to outdoor concentrations likely owing to the presence of open windows in the hospital. Mean ambient temperatures ranged from 20-34°C (mean = 27°C) on study days with relative humidity values ranging from 29-70% (mean = 49%). Twenty-four hour and 5-day mean concentrations of ambient PM_2.5_, NO_2_, and O_3_ were not strongly correlated with personal exposures during cycling (0.004 ≤ r ≤ 0.42). In addition, personal exposures to NO_2_, O_3_, and PM_2.5_ during cycling were only weakly correlated with regional concentrations of these pollutants during the same time period (0.02 ≤ r ≤ 0.18). Multi-pollutant models (including all pollutants) were selected for the final analyses as coefficients were often sensitive to the inclusion of other air pollutants (i.e. a change of more than 10%) (Additional file 1: Tables S2-S7). In addition to heart rate and temperature, final models included categorical variables for caffeine and alcohol consumption in the past 24-hours as these factors also had an important impact on model coefficients. Model estimates of the impact of traffic-related air pollution on RHI and blood pressure are shown in Figure 1. UFP exposures were associated with decreased micro-vascular function with each IQR increase in exposure corresponding to a 4.91% (95% CI: -9.31, -0.512) reduction in RHI values. Other air pollutants were not associated with reductions in micro-vascular function but each IQR increase in O_3_ was associated with a 2.49% (95% CI: 0.141, 4.84) increase in systolic blood pressure and a 3.26% (95% CI: 0.0117, 6.51) increase in diastolic blood pressure. Other air pollutants were not associated with changes in blood pressure. In general, similar associations were observed between UFPs and RHI and O_3_ and blood pressure in sensitivity analyses adjusted for potential carry-over effects of personal exposures in the preceding study period (Additional file 1: Table S8). Furthermore, adjusting RHI and blood pressure models for 24-hour or 5-day mean regional pollutant concentrations did not have a dramatic impact on model coefficients although some estimates were attenuated (Additional file 1: Table S8). In particular, adjustment for 5-day mean NO_2_ decreased the strength of the relationship between UFPs and RHI (mean change: -4.02%, 95% CI: -8.77, 0.72) and also decreased the relationship between O_3_ and diastolic blood pressure (mean change: 2.41%, 95% CI: -1.08, 5.91). However, adjusting for 24-hour mean O_3_ or 5-day mean NO_2_ resulted in statistically significant positive relationships between UFPs and diastolic blood pressure and also strengthened the association between O_3_ diastolic blood pressure (Additional file 1: Table S8). Additional adjustment for time between study days did not change the magnitude of the relationships between UFPs and RHI or O_3_ and blood pressure (less than a 10% change). Interaction terms between UFPs and 24-hour or 5-day mean regional NO_2_, O_3_, or PM_2.5_ were not statistically significant in RHI models (interaction p-values > 0.05). Likewise, interaction terms between O_3_ and 24-hour or 5-day mean regional NO_2_, O_3_, or PM_2.5_ were not statistically significant in blood pressure models (interaction p-values > 0.05). However, the relationship between O_3_ and blood pressure was stronger when 5-day mean regional PM_2.5_ was low (≤13.5 μg/m^3^) prior to study days (systolic mean change = 4.60%, 95% CI: -0.471, 9.68; diastolic mean change = 6.31%, 95% CI: -0.645, 13.3) compared to when regional PM_2.5_ values were above this median concentration (systolic mean change = 1.02%, 95% CI: -1.96, 4.00; diastolic mean change: 1.59%, 95% CI: -2.54, 5.71). Several pollutants were associated with changes in HRV over the 3-hour follow-up period (Figures 2 and 3). In particular, three pollutants were associated with increases in SDNN including O_3_ (mean change: 14.9 ms, 95% CI: 8.32, 21.6), black carbon (mean change: 3.56 ms, 95% CI: 0.865, 6.25), and UFPs (mean change: 3.61 ms, 95% CI: 0.227, 7.00). Conversely, each IQR increase in NO_2_ exposure was associated with a 5.92 ms (95% CI: -10.3, -1.50) decrease in SDNN although a similar decrease was not observed in the single-pollutant model. Personal PM_2.5_ exposures were inversely associated with HRV measures reflecting parasympathetic modulation of the heart. Specifically, each IQR increase in PM_2.5_ was associated with a 2.73 ms (95% CI: -4.73, -0.721) decrease in RMSSD, a 2.31% (95% CI: -3.96, -0.665) decrease in pNN50, and a 44.0 ms^2^ (95% CI: -107, 19.6) decrease in high-frequency power over the 3-hour follow-up period. A significant interaction was detected between O_3_ and black carbon in the multi-pollutant model for SDNN (p < 0.05) with a stronger positive association observed between O_3_ and SDNN at higher black carbon concentrations. In addition, a significant interaction was detected between O_3_ and NO_2_ in the multi-pollutant model for SDNN with a stronger association observed between O_3_ and SDDN at low NO_2_ levels (p < 0.05). In frequency domain analyses, O_3_ (mean change: 271 ms^2^, 95% CI: 100, 441) and NO_2_ (mean change: -187 ms^2^, 95% CI: -306, -67.8) were each associated with changes in LF over the 3-hour follow-up period although the direction of these associations differed. Ozone was also associated with an increase in the ratio of LF to HF in single pollutant models (mean change: 0.747, 95% CI: 0.0987, 1.40) but the magnitude of this relationship decreased and was no longer statistically significant in the multi-pollutant model (Figure 4). A significant interaction was not detected between NO_2_ and O_3_ in the model for LF (p > 0.05). When changes in HRV were examined at hourly intervals (i.e. lag 0-3 hours) the directions of associations were generally consistent across time points (Additional file 2: Figures S1-S2). The strongest association between black carbon and SDNN was observed 1-hour after exposure (mean change = 5.05 ms, 95% CI: 0.117, 10.0) whereas the inverse relationship between NO_2_ and SDNN was strongest immediately after exposure (mean change = -7.90, 95% CI: -15.9, 0.102). Relationships between UFPs, O_3_, and SDNN did not vary substantially across time points. Inverse relationships between PM_2.5_ and RMSSD (mean change = -4.09 ms, 95% CI: -7.94, -0.231), pNN50 (mean change = -3.34 ms, 95% CI: -6.53, -0.151), and HF (mean change = -69.9 ms^2^, 95% CI: -166, 25.8) were strongest 2-hours after exposure whereas relationships between O_3_ (mean change = 486 ms^2^, 95% CI: 114, 859), NO_2_ (mean change = -399 ms^2^, 95% CI: -672, -125), and LF were strongest 3-hours after exposure. Sensitivity analyses employing random coefficient models were generally consistent with the main analysis above although some differences were detected. Specifically, the relationship between UFPs and SDNN was stronger in random coefficient models (mean change: 9.86 ms, 95% CI: 0.245, 19.5) whereas associations with SDNN decreased for O_3_ (mean change: 5.27, 95% CI: -7.50, 18.0) and disappeared for black carbon (mean change: -0.465, 95% CI: -8.53, 7.60). In addition, a stronger inverse relationship was observed between PM_2.5_ and SDNN in random coefficient models (mean change: -12.8 ms, 95% CI: -28.3, 2.68). Inverse relationships between PM_2.5_ and HRV measures reflecting parasympathetic modulation remained consistent in random coefficient models (although decreased slightly for RMSSD and pNN50) and estimates describing the relationship between LF and NO_2_ and O_3_ were nearly identical to those in the main analysis. Further adjustment of HRV models for personal exposures during previous study periods or regional air quality did not have a meaningful impact on the magnitude or direction of coefficients from the main analysis reported in Figures 2 and 3 above (Additional file 1: Tables S9-S13). However, regional air pollutant concentrations did modify the impact of personal exposures on HRV (Additional file 1: S14-S16). In particular, regional PM_2.5_ concentrations 24-hours prior to each study period modified the impact of personal PM_2.5_ on HRV measures reflecting parasympathetic modulation (interaction p-values <0.05). Specifically, in stratified analyses each IQR increase in personal PM_2.5_ exposures was associated with significant decreases in RMSSD (mean change: -7.76 ms, 95% CI: -10.9, -4.58), pNN50 (mean change: -5.76%, 95% CI: -8.40, -3.12), and HF (mean change: -134 ms^2^, 95% CI: -234, -33.0) when 24-hour mean regional PM_2.5_ concentrations were above the median value (11.5 μg/m^3^) but non-significant positive associations were observed when regional PM_2.5_ concentrations were below this value (Figures 5 and 6). A similar pattern of effect modification was observed for interactions with 5-day mean regional PM_2.5_. Regional air pollutants also modified the effects of O_3_ and NO_2_ on HRV. Specifically, the inverse relationship between NO_2_ and SDNN was stronger when 5-day mean O_3_ concentrations were low (≤26.47 ppb) (mean change: -14.2 ms, 95% CI: -22.1, -6.68) compared to when regional values were above this value (mean change:-1.44 ms, 95% CI: -9.75, 6.87) (interaction p-value = 0.001) (Figure 7). Similarly, O_3_ was inversely associated with SDNN when 24-hour PM_2.5_ concentration were low (<11.5 μg/m^3^) (mean change: -28.7 ms, 95% CI: -44.1, -13.2) but was positively associated with SDDN when regional PM_2.5_ concentrations were above the median concentration (mean change: 24.0 ms, 95% CI: 15.7, 32.3) (Figure 7). A similar pattern of effect modification was observed for the relationship between O_3_ and SDNN with 5-day mean PM_2.5_ and NO_2_ (Figure 7). Ambient NO_2_ modified the impact of UFPs on SDNN (p = 0.015) with a stronger positive association observed when 24-hour NO_2_ concentrations were low (Additional file 1: Table S14). Similarly, ambient O_3_ modified (p = 0.008) the impact of black carbon on SDNN with a stronger positive association observed at low O_3_ concentrations (Additional file 1: Table S14). Regression coefficients for all models are available in the Additional Material (Additional file 1: Tables S2-S16).

**Table 1:** **Characteristics of the study population (n = 53)**

	**n**	**Mean/% (SD)**	**Range**
Age	53	25 (6.0)	18-44
Body Mass Index (kg/m^2^)	53	22 (2.9)	15-30
Racial Group			
Caucasian	29	54.7%	
Asian	13	24.5%	
Other	11	20.8%	
Second-Hand Smoke Exposure (Past 24-hours)			
Yes	19	13.3%	
No	124	86.7%	
Recent Illness in the past week			
Yes	13	9.1%	
No	130	90.9%	
Alcohol Consumption (Past 24-hours)			
None	109	76.2%	
1-5 Glasses	34	23.8%	
Caffeine Consumption (Past 24-hours)			
Yes	61	42.7%	
No	82	57.3%	
Regular Medication Use			
Yes	11	21%	
No	42	79%	

SD, standard deviation.

**Table 2:** **Baseline physiological measurements**

**Physiological measure**	**n**	**Mean/% (SD)**	**Range**
Heart Rate	142	78.5 (12)	53-105
Systolic Blood Pressure (mm Hg)	137	98. 4 (9.45)	80-129
Diastolic Blood Pressure (mm Hg)	137	62.1 (7.3)	44-80
Reactive Hyperemia Index	136	1.61 (0.31)	0.93-2.73
SDNN (ms)	142	112 (39)	43-229
RMSSD (ms)	142	28.3 (12)	9-78
pNN50 (%)	142	8.67 (8.5)	0-42
LF (ms^2^)	142	1316 (1189)	25-6764
HF (ms^2^)	142	296 (334)	11-1924
LF:HF	142	6.38 (5.0)	1.1-43

SD, standard deviation; SDNN, standard deviation of normal to normal intervals; RMSSD, root mean square of successive differences in adjacent NN intervals; pNN50, proportion of adjacent NN intervals differing by more than 50 ms; LF, low-frequency power; HF, high-frequency power.

**Table 3:** **Distribution of personal exposures to air pollution**

		**Overall**			**Route**		
**Air pollutant**	**n**	**Mean (SD)**	**Median (Range)**	**IQR**	**High-Traffic mean (SD)**	**Low-Traffic mean (SD)**	**Indoors mean (SD)**
*Personal Exposures*							
UFPs (count/cm^3^)	141	16,771 (10,302)	14,300 (3720-50,900)	9150-20,000	21,234 (9998)	18,740 (10,143)	9800 (6688)
Black Carbon (ng/m^3^)	140	1725 (1406)	1237 (33-8440)	914-2074	2260 (1692)	1248 (910)	1608 (1286)
PM_2.5_ (μg/m^3^)	143	14.2 (13)	8.63 (3.1-70.8)	4.2-19.4	15.7 (15.9)	13.4 (13.8)	13.4 (9.4)
NO_2_ (ppb)	136	48.0 (18)	46.8 (2.3-131)	35.2-57.0	48.9 (17.5)	50.9 (21.2)	43.3 (14.9)
O_3_ (ppb)	136	50.1 (16)	47.7 (17-101)	38.0-62.3	55.7 (12.1)	56.7 (17.2)	34.4 (7.9)
*Regional Air pollution Concentrations Prior to Study Periods*							
PM_2.5__5_ (μg/m^3^) (24 hours)	134	14.5 (10.9)	11.5 (1.88-52.9)	7.13-17.6			
NO_2_ (ppb) (24 hours)	143	7.02 (2.80)	6.41 (1.42-14.1)	5.05-8.34			
O_3_ (ppb) (24 hours)	143	26.9 (7.41)	26.4 (11.2-43.9)	21.1-31.6			
PM_2.5_ (μg/m^3^) (5 days)	132	14.5 (7.82)	13.5 (4.31-34.6)	7.63-20.2			
NO_2_ (ppb) (5 days)	143	6.53 (1.20)	6.59 (4.03-9.13)	5.71-7.51			
O_3_ (ppb) (5 days)	143	27.4 (5.24)	26.5 (16.1-38.5)	24.4-30.4			

UFP, ultrafine particles; IQR, interquartile range.

## Discussion

Air pollution is a known public health concern and is recognized as an important contributor to global disease burden [14]. In this study, we examined the impact of short-term exposures to traffic-related air pollution on physiological measures of cardiovascular function among healthy young women and noted several important results. In particular, our findings suggest that changes in blood pressure, cardiac autonomic modulation, and/or vasomotor function may play a role in explaining previous associations between short-term exposure to traffic and cardiovascular morbidity. Although the magnitudes of observed associations were small, our findings support the biological plausibility of a detrimental impact of traffic-related air pollution on cardiovascular health and may not be trivial for potentially sensitive sub-populations that are physically active in urban areas. In addition, our findings suggest that previous exposure to regional air pollution may modify the impact of traffic-related air pollution on cardiac autonomic modulation.

Endothelial dysfunction is an initiating event in the development of atherosclerosis [15,16], and in this study UFP exposures were associated with decreased RHI values 3-hours after exposure. This finding is consistent with a number of previous studies that have examined the impact of UFPs (and exposure to combustion exhaust which is a strong source of UFPs) on vasomotor function. Specifically, UFP exposures during exercise have previously been associated with decreases in brachial artery diameter and flow-mediated vasodilation among men [17,18] and several controlled exposure studies (primarily in men) have also noted inverse relationships between short-term UFP exposures and endothelial function [19-21]. Conversely, at least one other study did not observe an inverse relationship between UFPs and RHI values among healthy adults in a controlled exposure setting [22]; however, this discrepancy may be explained in part by lower exposure levels as mean exposures were approximately half those experienced on the high-traffic route in the current investigation. Regardless, toxicological data also supports an inverse relationship between UFPs and endothelial function as these pollutants have been shown to reduce the bioavailability of endothelial nitric oxide through scavenging by oxidative stress [23,24] or by direct action on endothelial nitric oxide synthase [25,26].

Gaseous pollutants were not associated with acute changes in endothelial function and this finding is consistent with previous studies of controlled human exposure to NO_2_ or O_3_ [27,28]. Conversely, while we did not observe an inverse relationship between PM_2.5_ and endothelial function among women, some evidence suggests that short-term exposure to high concentrations (~300 μg/m^3^) of PM_2.5_ from diesel exhaust may impair nitric-oxide mediated endothelial function by increasing oxidative stress [29]. Reasons for this discrepancy are not clear. One explanation may be related to differences in particle composition as the traffic fleet in Montreal is primarily composted of gasoline vehicles and thus PM_2.5_ emissions in this region may elicit a weaker response than particulate matter from diesel exhaust. However, controlled human exposure studies with PM_2.5_ concentrations similar to those in the present study have also failed to observe an important impact of short-term PM_2.5_ exposures on vascular function [22].

Increased sympathetic tone has been proposed as a plausible mechanism explaining the associations between air pollution and blood pressure [30] and our findings support this hypothesis. Specifically, personal exposure to ozone was associated with increases in both systolic and diastolic blood pressure 3-hours after exposure and the timing of this effect coincided with a positive association between ozone and low-frequency HRV, a parameter that at least partially reflects sympathetic modulation. In the main analysis, the positive association between O_3_ and SDNN appears to contradict this finding as increased sympathetic modulation is expected to decrease SDDN; however, findings from sensitivity analyses related to effect modification by regional air pollutants shed some light on this issue. Specifically, the positive relationships between O_3_ and blood pressure were strongest when regional PM_2.5_ was low and during these days O_3_ was inversely associated with SDNN and positively associated with LF which is consistent with increase sympathetic modulation (Additional file 1: Table S15). Therefore, our findings suggest that short-term exposure to ozone may increase blood pressure through increased sympathetic tone but that this relationship may be modified by previous exposure to PM_2.5_. Nevertheless, existing evidence related to the impact of ozone on blood pressure is somewhat inconsistent. For example, a controlled exposure study among men failed to observe an important impact of ozone on blood pressure 2-6 hours after exposure [28] and short-term exposure to ozone was not associated with blood pressure changes among cardiovascular disease patients in China [31]. Conversely, others have reported that ozone exposures in the first trimester may contribute to increases in blood pressure among women later in pregnancy [32] and long-term exposure to ozone has been associated with increased blood pressure among adults in China [33]. While other air pollutants were not associated with significant changes in blood pressure in this study, previous studies have reported positive relationships for PM_2.5_ [33,34] and black carbon [35]. However, these studies examined responses among cardiovascular disease patients [33,34] or subjects with metabolic syndrome [35] and thus differences in population susceptibilities may explain this discrepancy. Moreover, some evidence suggests that single nucleotide polymorphisms in microRNA processing genes may modify the relationship between short-term black carbon exposures and blood pressure [35]; therefore, genetic differences may also contribute to heterogeneity between studies.

In general, our findings suggest that several traffic pollutants may impact cardiac autonomic modulation among women in the hours immediately following exposure, although the directions of these impacts differed between pollutants. In particular, PM_2.5_ exposures were associated with decreases in HRV measures reflecting parasympathetic modulation and this is consistent with several recent studies of short-term personal exposure to traffic-related air pollution [36-39]; however, we did not observe similar associations in our previous study among cyclists [12]. Likewise, while personal UFP exposures were inversely associated with RMSSD and HF in at least two previous studies [12,36], similar relationships were not observed among women in Montreal. Reasons for this discrepancy are not entirely clear, but our sensitivity analysis of within-subject effects suggested possible heterogeneity in the direction of responses between individuals and future studies should explore potential explanations for these differences. For example, some evidence suggests that oxidant defence may modify the impact of PM_2.5_ on HRV [6] and this may be one factor contributing to heterogeneity in responses between subjects. In addition, obesity [40] may also modify the impact of air pollution on cardiovascular outcomes but it was not possible to evaluate this question in the present study as most women were not overweight or obese.

Few studies have examined the relationship between HRV and short-term personal exposure to NO_2_ or ozone. In general, our findings for NO_2_ are consistent with previous studies that have reported inverse associations between ambient NO_2_ and SDNN and/or LF [12,41,42]. However, some studies have failed to observe relationships between ambient NO_2_ and HRV [43,44] while others suggest that the impact of NO_2_ on HRV may be modified by disease status [45]. Personal exposure to ozone was positively associated with SDNN and LF among women in this study, but a recent panel study in Mexico City noted inverse associations between ozone and these outcomes [42]. Conversely, a controlled exposure study among men [28] did not observe an important impact of ozone on HRV at much higher exposure levels (300 ppb) than those experienced in the present study and two previous panel studies also failed to observe an association between ozone and HRV [43,46]. Finally, it is important to note that none of the pollutants examined in multi-pollutant models were associated with changes in the ratio of LF to HF, although evidence of a possible association was observed for ozone in single pollutant analyses. In general, this finding suggests that the balance of sympathetic/parasympathetic regulation was not altered by short-term exposure to traffic-pollution among women; however, such changes have been noted in previous studies [12,38].

We are not aware of other human studies that have examined effect modification by regional air pollution concentrations in assessing the short-term impacts of personal exposure to traffic-related air pollution on autonomic control of the heart. In general, our findings suggest that personal exposures to PM_2.5_ result in stronger decreases in parasympathetic modulation of the heart when regional PM_2.5_ concentrations are higher on the days prior to exposure. Regional PM_2.5_ also modified the impact of O_3_ on autonomic function and in general our results suggest that previous exposures to gaseous/particulate air pollutants may modify the acute impacts of more recent exposures on autonomic function. However, our findings with respect to effect modification should be interpreted with caution as regional monitors were shown to be poor measures of personal exposures in this study and thus it is not clear if regional concentrations actually represent personal exposures prior to participating in the study. Nevertheless, recent toxicological evidence suggests that ozone exposures may modify cardiovascular responses to PM_2.5_ and UFPs [47] and further evaluation of potential latent effects that modify air pollution impacts on autonomic function is warranted.

Our study had several strengths including a cross-over design to limit confounding by within-subject characteristics as well as detailed personal exposure monitoring for multiple air pollutants. Indeed, personal exposure monitoring is particularly important for pollutants with high spatial variability as exposure measurement error has been shown to mask associations between traffic-related air pollution and HRV [48]. Nevertheless, it is important to note several limitations. First, a large number of models were examined and it is possible that some associations may have occurred by chance. However, our main findings related to UFPs and RHI, O_3_ and blood pressure, and PM_2.5_/O_3_/NO_2_ and HRV were robust to a number of sensitivity analyses. Secondly, our findings reflect responses in healthy young women and may not be generalizable to subjects who may be more susceptible to air pollution health effects. In addition, the duration of follow-up after was short and the impact of traffic-related air pollution on blood pressure and endothelial function was only evaluated at a single time point following exposure. As a result, we do not know how long the observed effects might have persisted or if their magnitudes changed over time. Moreover, while heart rate was used to account for potential differences in exercise intensity (and thus minute ventilation) between exposure periods it is likely an imperfect measure; as a result, it possible that we did not complete account for these factors in our analyses. In addition, since all exposure periods involved exercise, we could not clearly differentiate the effects of physical activity from those of air pollution (although air pollution concentrations did vary substantially between exercise periods and exercise intensity was relatively constant between days). Therefore, it is important to recognize that our findings may represent the combined impact of both exercise and air pollution on the reported outcomes and future studies should include a control group without exercise but with exposure to address this limitation. In addition, we did not measure noise exposures during cycling and thus we could not account for this exposure in our analysis. This may be particularly important for HRV as some evidence suggests that noise may modify the impact of traffic-related air pollution on this outcome [31]. Future studies should evaluate this possibility further. While the use of an indoor cycling location was meant to increase variation in personal air pollution exposures, we noted similar pollutant concentrations between indoor and outdoor routes and between low and high-traffic routes for some pollutants on several study days. This may be viewed as a limitation as a lower exposure site would have increased statistical power; however, this also highlights the potential for infiltration of traffic pollutants in urban environments. Moreover, as we reported previously [12], the use of “high” and “low” routes based on traffic characteristics is not a suitable replacement for personal exposure measurements on any given day as high exposures can occur on “low” routes if strong sources (e.g. large diesel vehicles) are encountered. The use of a reactive hyperemia index to evaluate endothelial function may be viewed as a limitation as brachial artery flow mediated dilation is typically the gold standard measure of vasomotor function in clinical epidemiology [16]. However, the inverse relationship observed between baseline RHI and diastolic blood pressure among women supports the validity of this measure as previous studies have also reported inverse relationships between flow mediated dilation and blood pressure in healthy adults [49]. Finally, our study did not evaluate the impact of traffic-related air pollution on physiological measures during cycling, and thus we could not determine how short-term peaks in exposure may impact cardiovascular physiology during exercise. These factors should also be considered in future studies.

## Conclusions

Short-term exposure to traffic-related air pollution during physical activity has a measurable impact on physiological measures of cardiovascular function in healthy young women. In particular, our findings suggest that changes in blood pressure, cardiac autonomic modulation, and/or vasomotor function may contribute to previous associations between short-term exposure to traffic and cardiovascular morbidity. Moreover, our findings suggest that regional air pollution concentrations may modify the impact of subsequent exposures on autonomic function. While the observed associations were small in magnitude, they may be relevant for sensitive sub-populations that are physically active in urban areas.

### Methods study population

Women were recruited through advertisements posted at Montreal area colleges and universities as well as other public places in Montreal. Specifically, women were eligible to participate if they were non-smokers (and did not live with a smoker), were 18-45 years of age, and did not have a disease or disorder that prevented them from participating in moderate exercise on a bicycle over a continuous two-hour period. Women taking heart or anti-hypertensive medications were not eligible to participate in the study and pregnant or breast feeding women were also excluded. A telephone interview was conducted to make sure interested participants met the above eligibility criteria; medical screening was not conducted.

### Study design

A cross-over study design was used to evaluate the impact of short-term exposure to traffic-related air pollution on cardiovascular function in women. Specifically, women participated on three separate days (in random order) during which they cycled continuously over a two-hour period (between approximately 11:00-13:00) on either a high-traffic route (located in downtown Montreal), a low-traffic route (predominantly including park areas and residential roadways), or indoors (Additional file 2: Figure S3). Indoor cycling took place at the central study site located in a hospital in downtown Montreal. During indoor exercise, participants watched a video of a high-traffic route to mimic the starting and stopping action of outdoor exercise. Women were provided with water during cycling but did not eat during the exercise period. Visits were separated by at least 5-days (mean = 10 days) and up to two cyclists travelled along the same route on a given day along with one study technician as a safety precaution. All participants were asked to take the same route and mode of transportation to the study site each day in an effort to limit between-day differences in exposures prior to beginning the experiment each day. In addition, within-day differences (i.e. changes from baseline) in health measures were used to create outcome variables in order to limit the potential impacts of recent exposures (i.e. any such exposures would likely impact baseline and follow-up measures similarly given their close proximity in time). Questionnaires were used to collect demographic data as well as information on medication use, recent illness, alcohol/caffeine consumption, and recent exposure to environmental tobacco smoke. The institutional review boards of Health Canada and McGill University approved the study. Written informed consent was obtained from study participants prior to participation.

### Physiological measurements

Blood pressure, heart rate variability, and micro-vascular function were evaluated before and after each 2-hour exposure period. Follow-up measures for heart rate variability were collected immediately after exposure and each hour for 3-hours after exposure (lag 0-3 h) whereas micro-vascular function (reactive hyperemia index) and blood pressure were assessed 3-hours after exposure. The decision to limit tests of micro-vascular function and blood pressure to a single time-point following exposure was intended to minimize potential discomfort associated repeated occlusions of the brachial artery. All baseline physiological measures were collected at rest immediately prior to exposure and all measures were recorded in a quiet, dimly lit room with participants resting in a seated position.

Micro-vascular function was assessed using the non-invasive EndoPAT 2000 instrument (Itamar Medical Ltd, Cesari, Israel). This instrument measures endothelial-dependent (i.e. nitric-oxide mediated) vasodilation in the digital vasculature in response to flow mediated dilation induced by 5-minute occlusion of the brachial artery. Concurrent measurements collected from the digital vasculature of the occluded and non-occluded arms are used to determine a reactive hyperemia index (RHI) that is indicative of endothelial function [50]. Attenuation of this index is a sign of decreased endothelial function and has been shown to predict coronary endothelial dysfunction and adverse cardiovascular events in a clinical setting [51,52]. In particular, RHI values below 1.67 are indicative of endothelial dysfunction. This cut-off value is based on the correlation between RHI values and the gold standard method of assessing early coronary atherosclerosis [51]. Blood pressure was assessed using an automated oscillometric device (BP-True BPM100 monitors; BP-True Medical Devices, Coquitlam, BC, Canada). Three blood pressure measures were collected at each time-point (separated by approximately 30 seconds each) and the average of the two closest measures was used for analysis.

HRV data were collected using three-channel (seven-lead) digital Holter monitors (Seer Light Extend, GE Medical Systems Information Technologies Inc, Milwaukee, Wi, USA) with subsequent analysis on a MARS workstation (version 7.2). Beat annotations were automatically assigned by the software and were verified and reviewed by technicians at the Arrhythmia Monitoring Center in Ottawa, Canada. Only normal sinus beats were used in the analysis. Time domain (SDNN, standard deviation of normal-to-normal intervals; RMSSD, root mean square of successive differences in adjacent NN intervals; pNN50, proportion of adjacent NN intervals differing by more than 50 ms) and frequency domain (LF, low frequency power (0.04-0.15 Hz); HF, high frequency power (0.15-0.40 Hz)) measures of HRV were based on the last 5-mintutes of each segment of the study day (baseline, immediately following exposure, and 1-3 hours after exposure) according to recommended standards [53]. Mean heart rate during cycling was also determined from Holter monitors to account for potential differences in exercise intensity across study days. Of the HRV parameters examined, RMSSD, pNN50 and HF reflect parasympathetic modulation of the heart, LF reflects a mixture of both sympathetic and parasympathetic modulation, and SDNN reflects overall heart rate variability [53]. The ratio of LF to HF is thought to reflect the balance of sympathetic and parasympathetic modulation.

### Air pollution measurements

Personal exposures to traffic-related air pollutants were measured using instruments mounted on bicycle panniers carried as previously described [12]. Specifically, concentrations of fine (particles with aerodynamic diameters less than 2.5 μm; PM_2.5_) and ultrafine particulate matter (UFPs) (0.01-0.1μm in diameter) were determined using Harvard Impactors (10 L/minute) and TSI Model 3007 instruments, respectively (TSI, St Paul, MN, USA). The instrument used to monitor UFPs measures the number concentration of particles (i.e. particles/cm^3^) as particle numbers are dominated by the ultrafine fraction of particulate air pollution. Black carbon exposures were determined using MicroAeth Model AE51 aethalometers (Magee Scientific, Berkeley, CA, USA) and NO_2_ and O_3_ exposures were measured using Ogawa sampling badges (Ogawa and Company, FL, USA) with subsequent analysis by ion chromatography. Real-time ambient temperature data were also recorded during each exercise period using HOBO Data Loggers (Onset, Cape Cod, MA, USA). Mean pollutant concentrations during each 2-hour exercise period were the primary exposure variables examined in statistical models.

### Statistical analysis

Linear mixed-effects models with random-subject intercepts were used to estimate the impact of air pollution exposures on physiological measures. All models included continuous measures of mean heart rate during exercise to account for potential differences in exercise intensity between routes. In addition, all models included a linear term for continuous measures of mean temperature during exercise to account for the potential impact of thermal stress on physiological measures. Other factors examined as potential covariates included caffeine consumption in the past 24-hours (yes/no), alcohol consumption in the past 24-hours (4-categories), race, age, body mass index (BMI), recent illness (yes/no), and second hand smoke exposure in the past 24-hours (yes/no). These variables were only included in final models if they had a meaningful impact (a change of 10% or more) on model coefficients. We did not examine potential effect modification by BMI as few participants (n = 13) were classified as overweight or obese (BMI > 25 kg/m^2^).

Single and multi-pollutant models (including all air pollutants) were examined for each outcome. For RHI and blood pressure, the dependent variable was the percent change from the baseline value 3-hours after exposure ((follow-up – baseline/baseline) × 100%). Models for percent changes in HRV were also examined but model fit was better when the outcome was modelled as the difference between follow-up and baseline measurements based on the Akaike Information Criterion (AIC) and Bayesian Information Criterion (BIC). Therefore, HRV models examined the impact of traffic-related air pollutants on the difference between pre and post-exposure measurements (follow-up-baseline). Changes in physiological measurements were approximately normally distributed for each outcome. If two pollutants were associated with the same outcome potential interactions were assessed by including the appropriate first order interaction term in the model. For HRV, separate models were examined for each time-period (lag 0-3h) as well as over the entire 3-hour follow-up period by combining measurements in a single mixed model for each outcome. As sensitivity analysis, random coefficient models with subject-specific slopes (for air pollutants) and intercepts were also examined for HRV over the entire follow-up period. Only one random coefficient (for a given air pollutant) was included in the model at a time with all other covariates modeled as fixed effects. These models assumed an unstructured covariance structure between the random slopes and intercepts. As relatively few data points were available to estimate subject-specific slopes for air pollutant impacts on HRV, the purpose this analysis was to determine if the magnitude and direction of regression coefficients remained stable when slopes were allowed to vary between subjects. Subject-specific scatter plots were also examined to evaluate consistency in the direction of air pollution effects on HRV across participants.

Sensitivity analyses were conducted to adjust for potential carry-over effects from previous visits as well regional air quality in the days prior to each study period. Specifically, in order to evaluate potential carry-over effects, continuous terms for exposures experienced during the previous visit were added to the models described above (the first visit was assigned a value of zero since there was no previous visit). In this analysis, each previous exposure was evaluated separately (i.e. only one previous exposure was examined at a time). In addition, we examined models including a term for the number of days between visits to evaluate how this parameter may influence model coefficients. A similar approach was used to evaluate the impact of regional air quality on the reported outcomes. Specifically, the main models described above were additionally adjusted for continuous measures of 24-hour or 5-day mean regional ambient concentrations of NO_2_, O_3_, or PM_2.5_ (separately) prior each exposure period. Regional air quality data were available from a fixed site station in Montreal. Finally, if personal exposure to a given pollutant was associated with changes in RHI, blood pressure, or HRV in the main analyses, potential interactions with regional air pollutants were evaluated by including the appropriate first-order interaction term in the models above. If a statistically significant interaction was detected (p < 0.05), stratified models were also examined using the median value of regional air pollution concentrations as the cut point. All coefficients reflect interquartile range (IQR) changes in mean UFP (10,850/cm^3^), black carbon (1160 ng/m^3^), PM_2.5_ (15.2 μg/m^3^), NO_2_ (21.7 ppb), and O_3_ (24.2 ppb) exposures during exercise. Analyses were not conducted by “route” (i.e. high/low/indoors) as previous evidence suggests that high personal exposures can occur on “low” routes and thus broad classification of exposures based on these criteria likely contribute to exposure misclassification [12]. All statistical analyses were conducted using Stata/MP (xtmixed procedure) (v11; StataCorp LP, USA) and R (version 2.15; R Core Team).

## Additional files

Additional file 1: Figure S1. Scatter plot of baseline RHI and baseline diastolic blood pressure. **Table S1:** Spearman correlations between pollutants; **Table S2:** Single-pollutant models for the relationship between air pollutants and changes in reactive hyperemia index and blood pressure; **Table S3:** Multi-pollutant models for the relationship between air pollutants and changes in reactive hyperemia index and blood pressure; **Table S4:** Single-Pollutant Models for the Relationship between Personal Air Pollution Exposures and Acute Changes in HRV over the entire follow-up period. **Table S5:** Multi-Pollutant Models for the Relationship between Personal Air Pollution Exposures and Acute Changes in HRV over the entire follow-up period. **Table S6:** Random-Coefficient Multi-Pollutant Models for the Relationship between Personal Air Pollution Exposures and Acute Changes in HRV over the entire follow-up period. **Table S7:** Multi-Pollutant Models for the Relationship between Personal Air Pollution Exposures and Acute Changes in HRV at Hourly Intervals following Exposure. **Table S8:** Multi-Pollutant Models for the Relationship between Personal Air Pollution Exposures and Percent Changes in Reactive Hyperemia Index and Blood Pressure 3-hours after Exercise Adjusted for Exposures during Previous visits and Regional Air Quality. **Tables S9-S13:** Multi-Pollutant Models for the Relationships between Personal exposures to UFPs (S9), black carbon (S10), PM_2.5_ (S11), NO_2_ (S12), and O_3_ (S13) and Acute Changes in HRV over the entire follow-up period with additional adjustment for exposures during previous visits and regional air quality. **Table S14:** Personal Exposure to PM_2.5_, UFPs, and Black Carbon and Effect Modification by Regional Air Pollution in HRV models. **Table S15:** Personal Exposure to O_3_ and Effect Modification by Regional Air Pollution in HRV models. **Table S16:** Personal Exposure to NO_2_ and Effect Modification by Regional Air Pollution in HRV models. Additional file 2: Figure S1. Relationship between personal air pollution exposures and hourly changes in time-domain measures of HRV. **Figure S2:** Relationship between personal air pollution exposures and hourly changes in frequency-domain measures of HRV. **Figure S3:** Map of Cycling Routes.

## Abbreviations

AIC: Akaike information criterion
BIC: Bayesian information criterion
BMI: Body mass index
HF: High-frequency power
HRV: Heart rate variability
IQR: Interquartile range
LF: Low-frequency power
pNN50: Proportion of adjacent NN intervals differing by more than 50 ms
RHI: Reactive hyperemia index
RMSSD: Root mean square of successive differences in adjacent NN intervals
SD: Standard deviation
SDNN: Standard deviation of normal to normal intervals
UFP: Ultrafine particles
