# Supplementary material for: Exposure to traffic-related air pollution during physical activity and acute changes in blood pressure, autonomic and micro-vascular function in women: a cross-over study
Source: Part Fibre Toxicol. 2014 Dec 9;11:70. doi: 10.1186/s12989-014-0070-4 (PMC4276095; doi:10.1186/s12989-014-0070-4)
Supplement: Additional file 1: Figure S1. — Scatter plot of baseline RHI and baseline diastolic blood pressure. Table S1: Spearman correlations between pollutants; Table S2: Single-pollutant models for the relationship between air pollutants and changes in reactive hyperemia index and blood pressure; Table S3: Multi-pollutant models for the relationship between air pollutants and changes in reactive hyperemia index and blood pressure; Table S4: Single-Pollutant Models for the Relationship between Personal Air Pollution Exposures and Acute Changes in HRV over the entire follow-up period. Table S5: Multi-Pollutant Models for the Relationship between Personal Air Pollution Exposures and Acute Changes in HRV over the entire follow-up period. Table S6: Random-Coefficient Multi-Pollutant Models for the Relationship between Personal Air Pollution Exposures and Acute Changes in HRV over the entire follow-up period. Table S7: Multi-Pollutant Models for the Relationship between Personal Air Pollution Exposures and Acute Changes in HRV at Hourly Intervals following Exposure. Table S8: Multi-Pollutant Models for the Relationship between Personal Air Pollution Exposures and Percent Changes in Reactive Hyperemia Index and Blood Pressure 3-hours after Exercise Adjusted for Exposures during Previous visits and Regional Air Quality. Tables S9-S13: Multi-Pollutant Models for the Relationships between Personal exposures to UFPs (S9), black carbon (S10), PM2.5 (S11), NO2 (S12), and O3 (S13) and Acute Changes in HRV over the entire follow-up period with additional adjustment for exposures during previous visits and regional air quality. Table S14: Personal Exposure to PM2.5, UFPs, and Black Carbon and Effect Modification by Regional Air Pollution in HRV models. Table S15: Personal Exposure to O3 and Effect Modification by Regional Air Pollution in HRV models. Table S16: Personal Exposure to NO2 and Effect Modification by Regional Air Pollution in HRV models. [file 12989_2014_70_MOESM1_ESM.docx]

ADDITIONAL FILE 1

Exposure to Traffic-Related Air Pollution during Physical Activity and Acute changes in Blood Pressure, Autonomic and Micro-Vascular Function in Women: a Cross-Over Study

Scott Weichenthal*^1^* *Corresponding author Email: [scott.weichenthal@hc-sc.gc.ca](mailto:scott.weichenthal@hc-sc.gc.ca)

Marianne Hatzopoulou^2^ Email: marianne.hatzopoulou@mcgill.ca

Mark S. Goldberg^3^ Email: [mark.goldberg@mcgill.ca](mailto:mark.goldberg@mcgill.ca)

^1^ Air Health Science Division, Health Canada, 269 Laurier Avenue West, K1A 0K9, Ottawa, ON, Canada.

^2^ Department of Civil Engineering, McGill University, Macdonald Engineering Building
817 Sherbrooke Street West, H3A 0C3, Montreal, Quebec, Canada.

^3^ Division of Clinical Epidemiology, McGill University Health Center, 687 Pine Avenue West, H3A 1A1, Montreal, Quebec, Canada.

Figure S1. **Scatter plot of baseline RHI and baseline diastolic blood pressure**. Line and shaded area reflect the slope and 95% confidence interval, respectively


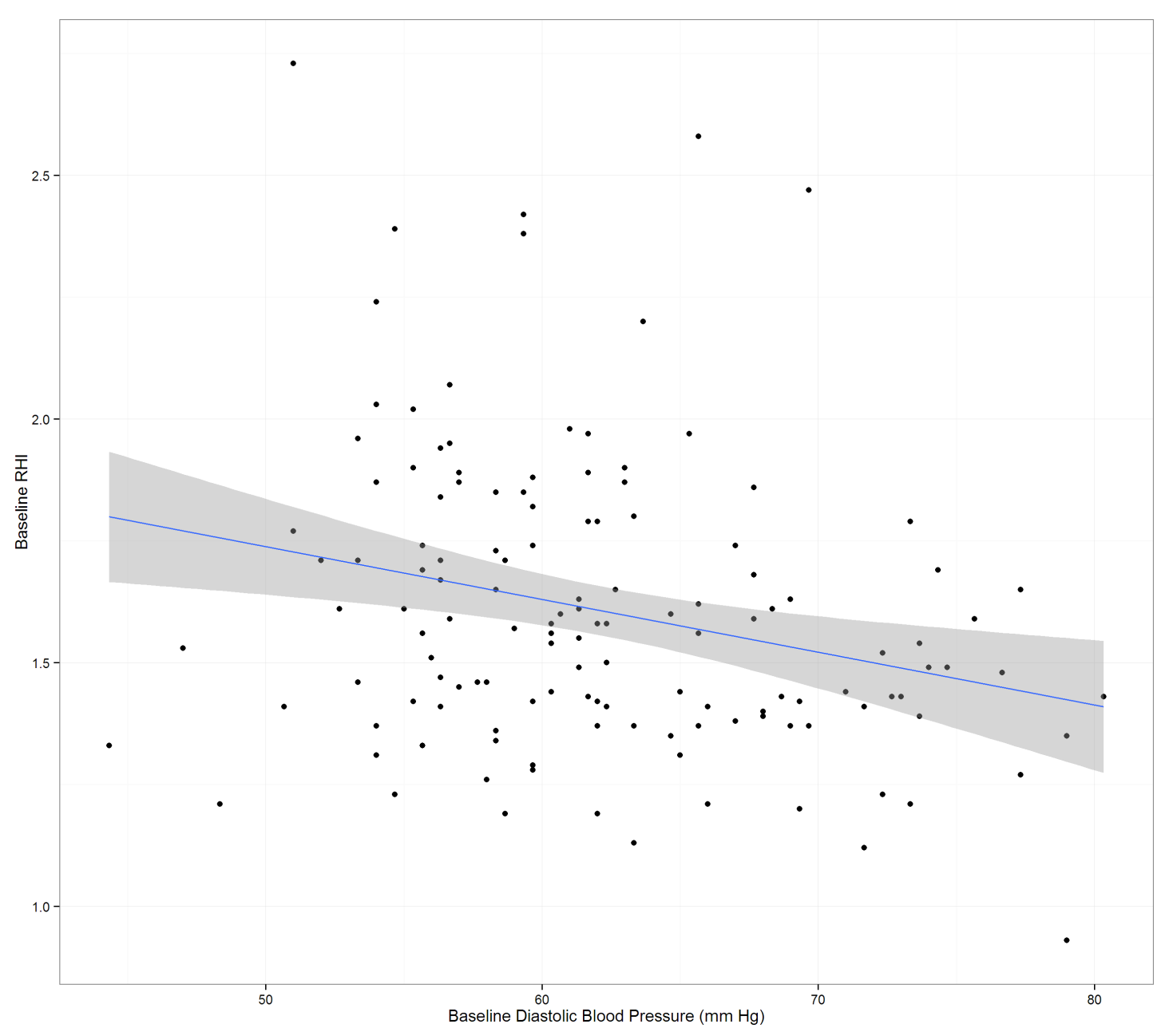


Table S1. Spearman correlations between personal air pollution exposures

|  | UFP | Black Carbon | PM_2.5_ | NO_2_ |
| --- | --- | --- | --- | --- |
| UFP | 1 |  |  |  |
| Black Carbon | 0.26 | 1 |  |  |
| PM_2.5_ | 0.080 | 0.13 | 1 |  |
| NO_2_ | 0.17 | 0.21 | 0.043 | 1 |
| O_3_ | 0.24 | 0.16 | 0.048 | 0.33 |

UFP, ultrafine particles.

Table S2. Single-Pollutant Models for the Relationship between Personal Air Pollution Exposures and Percent Changes in Reactive Hyperemia Index and Blood Pressure 3-hours after Exercise

| Exposure | Percent Change in Outcome | | |
| --- | --- | --- | --- |
|  | RHI  β (95% CI) | Systolic BP  β (95% CI) | Diastolic BP  β (95% CI) |
| UFPs | -4.63  (-8.57, -0.693) | 0.372 (-0.816, 1.56) | 1.29 (-0.329, 2.91) |
| Black Carbon | -0.280  (-3.38, 2.82) | -0.212 (-1.13, 0.701) | -0.0970 (-1.36, 1.16) |
| PM_2.5_ | 1.56  (-2.89, 6.02) | 0.358 (-0.970, 1.69) | -0.717 (-2.54, 1.11) |
| NO_2_ | -1.07  (-5.77, 3.64) | 0.300 (-1.05, 1.65) | -0.400 (-2.32, 1.52) |
| O_3_ | -1.86  (-8.07, 4.35) | 1.42 (-0.376, 3.22) | 2.50 (-0.0362, 5.03) |

RHI, reactive hyperaemia index; BP, blood pressure; UFP, ultrafine particles. All models are adjusted for ambient temperature during exercise, mean heart rate during exercise, and alcohol/caffeine consumption in the past 24-hours. Regression coefficients reflect interquartile range increases in exposure.

Table S3. Multi-Pollutant Models for the Relationship between Personal Air Pollution Exposures and Percent Changes in Reactive Hyperemia Index and Blood Pressure 3-hours after Exercise

| Exposure | Percent Change in Outcome | | |
| --- | --- | --- | --- |
|  | RHI  β (95% CI) | Systolic BP  β (95% CI) | Diastolic BP  β (95% CI) |
| UFPs (count/cm^3^) | **-**4.91  (-9.31, -0.512) | 0.377 (-0.900, 1.65) | 1.61 (-0.155, 3.38) |
| Black Carbon (ηg/m^3^) | 0.546  (-2.84, 3.94) | -0.264 (-1.26, 0.734) | -0.233 (-1.61, 1.15) |
| PM_2.5_ (µg/m^3^) | 4.11  (-2.09, 10.3) | 0.775 (-1.08, 2.63) | -1.31 (-3.88, 1.25) |
| NO_2_ (ppb) | -1.52  (-7.31, 4.28) | -0.559 (-2.26, 1.14) | -1.35 (-3.70, 0.991) |
| O_3_ (ppb) | 1.26  (-6.66, 9.18) | 2.49 (0.141, 4.84) | 3.26 (0.0117, 6.51) |

RHI, reactive hyperaemia index; BP, blood pressure; UFP, ultrafine particles. All models are adjusted for ambient temperature during exercise, mean heart rate during exercise, and alcohol/caffeine consumption in the past 24-hours. Regression coefficients reflect interquartile range increases in exposure and are mutually adjusted for all other air pollutants.

Table S4. Single-Pollutant Models for the Relationship between Personal Air Pollution Exposures and Acute Changes in HRV over the entire follow-up period

|  | Heart Rate Variability | | | | | |
| --- | --- | --- | --- | --- | --- | --- |
|  | SDNN (ms)  β (95% CI) | RMSSD (ms)  β (95% CI) | pNN50 (%)  β (95% CI) | LF (ms^2^)  β (95% CI) | HF (ms^2^)  β (95% CI) | LF:HF  β (95% CI) |
| UFP | 4.05 (0.820, 7.28) | 0.786 (-0.455, 2.03) | 0.543 (-0.492, 1.58) | 47.4 (-38.3, 133) | 25.7 (-12.8, 64.1) | -0.00942 (-0.357, 0.338) |
| BC | 2.27 (-0.127, 4.66) | -0.0104 (-0.945, 0.925) | 0.558 (-0.725, 0.836) | 61.8 (-1.94, 126) | 8.27 (-20.8, 37.4) | 0.0259 (-0.279, 0.331) |
| PM_2.5_ | 2.12 (-1.52, 5.77) | -1.58 (-2.96, -0.201) | -1.38 (-2.53, -0.237) | 43.8 (-50.8, 138) | -26.2 (-69.3, 16.8) | 0.226 (-0.224, 0.676) |
| NO_2_ | -0.490 (-4.25, 3.27) | 0.411 (-1.02, 1.84) | 0.431 (-0.747, 1.61) | -71.7 (-170, 26.3) | -9.84 (-54.8, 35.2) | -0.201 (-0.668, 0.266) |
| O_3_ | 9.75 (4.49, 15.0) | 1.56 (-0.426, 3.55) | 1.17 (-0.458, 2.79) | 125 (-9.88, 259) | -10.6 (-72.9, 51.7) | 0.747 (0.0987, 1.40) |

UFP, ultrafine particles. All models are adjusted for ambient temperature during exercise, mean heart rate during exercise, and alcohol/caffeine consumption in the past 24-hours. Regression coefficients reflect interquartile range increases in exposure.

Table S5. Multi-Pollutant Models for the Relationship between Personal Air Pollution Exposures and Acute Changes in HRV over the entire follow-up period

|  | Heart Rate Variability | | | | | |
| --- | --- | --- | --- | --- | --- | --- |
|  | SDNN (ms)  β (95% CI) | RMSSD (ms)  β (95% CI) | pNN50 (%)  β (95% CI) | LF (ms^2^)  β (95% CI) | HF (ms^2^)  β (95% CI) | LF:HF  β (95% CI) |
| UFP | 3.61 (0.227, 7.00) | 0.920 (-0.442, 2.28) | 0.680 (-0.445, 1.81) | 60.3 (-32.8, 153) | 24.7 (-18.5, 67.9) | 0.0445 (-0.337, 0.426) |
| BC | 3.56 (0.865, 6.25) | -0.460 (-1.53, 0.615) | -0.483 (-1.37, 0.403) | 66.3 (-6.48, 139) | 9.52 (-24.5, 43.6) | 0.0919 (-0.209, 0.392) |
| PM_2.5_ | -1.24 (-6.32, 3.84) | -2.73 (-4.73, -0.721) | -2.31 (-3.96, -0.665) | 37.3 (-97.6, 172) | -44.0 (-107, 19.6) | -0.00579 (-0.570, 0.559) |
| NO_2_ | -5.92 (-10.3, -1.50) | 0.347 (-1.41, 2.10) | 0.553 (-0.89, 2.00) | -187 (-306, -67.8) | -8.85 (-64.5, 46.8) | -0.401 (-0.895, 0.0935) |
| O_3_ | 14.9 (8.32, 21.6) | 2.23 (-0.325, 4.78) | 1.47 (-0.623, 3.56) | 271 (100, 441) | -0.992 (-81.5, 79.6) | 0.200 (-0.535, 0.936) |

UFP, ultrafine particles. All models are adjusted for ambient temperature during exercise, mean heart rate during exercise, and alcohol/caffeine consumption in the past 24-hours. Regression coefficients reflect interquartile range increases in exposure and are mutually adjusted for all other air pollutants.

Table S6. Random-Coefficient Multi-Pollutant Models for the Relationship between Personal Air Pollution Exposures and Acute Changes in HRV over the entire follow-up period

|  | Heart Rate Variability | | | | | |
| --- | --- | --- | --- | --- | --- | --- |
|  | SDNN (ms)  β (95% CI) | RMSSD (ms)  β (95% CI) | pNN50 (%)  β (95% CI) | LF (ms^2^)  β (95% CI) | HF (ms^2^)  β (95% CI) | LF:HF  β (95% CI) |
| UFP | 9.86 (0.245, 19.5) | 2.06 (-0.485, 4.61) | 1.42 (-0.660, 3.50) | -16.5 (-192, 159) | 19.7 (-33.4, 72.7) | -0.324 (-1.58, 0.931) |
| BC | -0.465 (-8.53, 7.60) | -1.33 (-3.92, 1.26) | -0.945 (-2.96, 1.07) | 89.2 (-62.9, 241) | -6.06 (-58.9, 46.8) | 0.129 (-0.805, 1.06) |
| PM_2.5_ | -12.8 (-28.3, 2.68) | -1.60 (-5.08, 1.87) | -1.22 (-4.00, 1.57) | 31.9 (-131, 195) | -38.5 (-122, 44.7) | -0.967 (-2.70, 0.771) |
| NO_2_ | 1.06 (-7.77, 9.89) | 0.378 (-2.61, 3.37) | 0.213 (-2.19, 2.62) | -189 (-371, -6.95) | -9.21 (-68.9, 50.5) | -0.114 (-1.29, 1.06) |
| O_3_ | 5.27 (-7.50, 18.0) | 0.607 (-3.32, 4.53) | 0.545 (-2.39, 3.48) | 267 (-13.2, 547) | -10.2 (-97.6, 77.3) | 0.140 (-1.01, 1.29) |

UFP, ultrafine particles. All models are adjusted for ambient temperature during exercise, mean heart rate during exercise, and alcohol/caffeine consumption in the past 24-hours. Regression coefficients reflect interquartile range increases in exposure and are mutually adjusted for all other air pollutants.

Table S7. Multi-Pollutant Models for the Relationship between Personal Air Pollution Exposures and Acute Changes in HRV at Hourly Intervals following Exposure

|  | Lag | Heart Rate Variability | | | | | |
| --- | --- | --- | --- | --- | --- | --- | --- |
|  |  | SDNN (ms)  β (95% CI) | RMSSD (ms)  β (95% CI) | pNN50 (%)  β (95% CI) | LF (ms^2^)  β (95% CI) | HF (ms^2^)  β (95% CI) | LF:HF  β (95% CI) |
| UFP | 0-h  1-h  2-h  3-h | 1.79 (-4.52, 8.10)  1.54 (-4.79, 7.87)  3.50 (-3.47, 10.5)  3.17 (-4.14, 10.5) | 0.925 (-1.19, 3.04)  0.691 (-2.03, 3.41)  0.799 (-1.37, 2.96)  1.50 (-1.02, 4.03) | 0.686 (-1.04, 2.41)  0.140 (-2.12, 2.40)  0.721 (-1.02, 2.47)  1.13 (-1.00, 3.25) | 34.0 (-117, 185)  75.5 (-84.3, 235)  -56.7 (-204, 90.2)  104 (-116, 322) | 20.5 (-35.6, 76.5)  -10.4 (-79.2, 58.4)  1.22 (-76.0, 78.5)  82.7 (-3.49, 169) | -0.298 (-1.07, 0.479)  0.0929 (-0.596, 0.782)  -0.157 (-0.889, 0.575)  0.272 (-0.481, 1.02) |
| BC | 0-h  1-h  2-h  3-h | 4.77 (-0.136, 9.68)  5.05 (0.117, 10.0)  3.72 (-1.73, 9.17)  -0.775 (-6.46, 4.91) | 0.711 (-0.940, 2.36)  -0.700 (-2.81, 1.41)  -1.53 (-3.23, 0.167)  -0.154 (-2.11, 1.80) | 0.519 (-0.822, 1.86)  -0.555 (-2.31, 1.20)  -1.74 (-3.12, -0.375)  0.135 (-1.51, 1.78) | 107 (-9.75, 224)  111 (-14.0, 235)  6.71 (-106, 119)  34.6 (-133, 202) | 49.8 (5.91, 93.6)  -3.97 (-57.5, 49.5)  -4.28 (-64.8, 56.2)  -23.2 (-90.1, 43.7) | -0.108 (-0.707, 0.492)  0.250 (-0.279, 0.780)  0.131 (-0.431, 0.694)  0.0997 (-0.481, 0.681) |
| PM_2.5_ | 0-h  1-h  2-h  3-h | -0.322 (-9.30, 8.66)  -4.46 (-13.4, 4.59)  0.261 (-9.79 , 10.3)  6.92 (-3.50, 17.3) | -0.666 (-3.69, 2.36)  -4.09 (-7.94, -0.231)  -2.43 (-5.58, 0.720)  -0.742 (-4.30, 2.82) | -0.698 (-3.15, 1.75)  -3.34 (-6.53, -0.151)  -1.94 (-4.48, 0.597)  -0.733 (-3.72, 2.26) | 85.1 (-126, 296)  -17.5 (-247, 212)  53.7 (-148, 256)  199 (-104, 502) | 27.2 (-53.6, 108)  -69.9 (-166, 25.8)  -110 (-221, 1.82)  -20.4 (-144, 103) | 0.302 (-0.790, 1.39)  -0.0710 (-1.03, 0.890)  -0.0923 (-1.11, 0.927)  -0.0484 (-1.12, 1.02) |
| NO_2_ | 0-h  1-h  2-h  3-h | -7.90 (-15.9, 0.102)  -5.79 (-13.8, 2.26)  0.248 (-8.65, 9.14)  -5.52 (-14.7, 3.69) | -1.45 (-4.14, 1.25)  0.645 (-2.80, 4.09)  2.04 (-0.739, 4.82)  -1.23 (-4.40, 1.94) | -1.32 (-3.51, 0.865)  0.572 (-2.28, 3.43)  2.19 (-0.0528, 4.43)  -0.193 (-2.86, 2.47) | -163 (-354, 28.6)  -95.9 (-299, 107)  -41.2 (-224, 142)  -399 (-672, -125) | -54.3 (-126, 17.3)  -10.5 (-96.6, 75.6)  73.0 (-25.7, 172)  -82.4 (-191, 26.7) | 0.171 (-0.807, 1.15)  -0.329 (-1.19, 0.536)  0.0215 (-0.897, 0.940)  -0.569 (-1.52, 0.379) |
| O_3_ | 0-h  1-h  2-h  3-h | 7.05 (-4.10, 18.2)  7.97 (-3.31, 19.2)  6.98 (-5.58, 19.5)  8.27 (-4.79, 21.3) | 2.75 (-1.01, 6.52)  2.05 (-2.73, 6.84)  1.89 (-2.06, 5.86)  3.26 (-1.18, 7.70) | 2.48 (-0.565, 5.52)  1.16 (-2.79, 5.11)  0.912 (-2.27, 4.10)  2.56 (-1.17, 6.29) | 151 (-110, 413)  228 (-57.2, 513)  18.1 (-234, 270)  486 (114, 859) | 44.4 (-56.5, 145)  3.36 (-115, 121)  -15.9 (-155, 124)  18.4 (-137, 173) | 0.764 (-0.602, 2.13)  -0.144 (-1.35, 1.06)  -0.512 (-1.79, 0.763)  0.0695 (-1.28, 1.42) |

UFP, ultrafine particles; BC, black carbon. All models are adjusted for ambient temperature during exercise, mean heart rate during exercise, and alcohol/caffeine consumption in the past 24-hours. Regression coefficients reflect interquartile range increases in exposure and are mutually adjusted for all other air pollutants.

Table S8. Multi-Pollutant Models for the Relationship between Air Pollution Exposures and Percent Changes in Reactive Hyperemia Index and Blood Pressure 3-hours after Exercise with Additional Adjustment for Exposures during Previous Visits and Regional Air Quality

| Exposure | Percent Change in Outcome | | |
| --- | --- | --- | --- |
|  | RHI  β (95% CI) | Systolic BP  β (95% CI) | Diastolic BP  β (95% CI) |
| UFPs^a^ | -4.91 (-9.31, -0.51) | 0.377 (-0.900, 1.65) | 1.61 (-0.155, 3.38) |
| Adjusted (separately) for: | |  |  |
| Previous Exposures^b^: |  |  |  |
| UFPs (count/cm^3^) | -5.67 (-10.1, -1.2) | 0.655 (-0.655, 1.96) | 2.18 (0.384, 3.97) |
| Black Carbon (ηg/m^3^) | -5.25 (-9.69, -0.82) | 0.572 (-0.690, 1.83) | 1.83 (0.051, 3.62) |
| PM_2.5_ (µg/m^3^) | -4.74 (-9.21, -0.26) | 0.421 (-0.862, 1.70) | 1.65 (-0.115, 3.42) |
| NO_2_ (ppb) | -5.03 (-9.52, -0.54) | 0.590 (-0.683, 1.86) | 2.00 (0.253, 3.74) |
| O_3_ (ppb) | -4.62 (-9.07, -0.168) | 0.565 (-0.698, 1.83) | 1.88 (0.126, 3.64) |
| Time between study days | -4.97 (-9.41, -0.53) | 0.538 (-0.713, 1.79) | 1.74 (-0.031, 3.51) |
| Regional Air Quality^c^ | |  |  |
| 24-hour mean PM_2.5_ | -4.71 (-10.8, 1.38) | 0.274 (-1.57, 2.12) | 2.26 (-0.237, 4.76) |
| 24-hour mean NO_2_ | -4.89 (-9.31, -0.47) | 0.341 (-0.940, 1.62) | 1.59 (-0.190, 3.37) |
| 24-hour mean O_3_ | -4.78 (-9.38, -0.18) | 0.456 (-0.883, 1.79) | 2.19 (0.375, 4.00) |
| 5-day mean PM_2.5_ | -4.07 (-10.1, 1.97) | 0.377 (-1.42, 2.18) | 2.41 (-0.076, 4.89) |
| 5-day mean NO_2_ | -4.02 (-8.77, 0.72) | 0.524 (-0.848, 1.90) | 2.04 (0.160, 3.92) |
| 5-day mean O_3_ | -4.79 (-9.39, -0.18) | 0.0953 (-1.22, 1.41) | 1.73 (-0.117, 3.57) |
| O_3_^a^ | 1.26 (-6.66, 9.18) | 2.49 (0.141, 4.84) | 3.26 (0.0117, 6.51) |
| Adjusted (separately) for: |  |  |  |
| Previous Exposures^b^: |  |  |  |
| UFPs (count/cm^3^) | -0.087 (-8.17, 8.00) | 2.45 (0.058, 4.85) | 3.56 (0.277, 6.84) |
| Black Carbon (ηg/m^3^) | 1.80 (-6.17, 9.77) | 2.53 (0.212, 4.85) | 3.28 (0.027, 6.54) |
| PM_2.5_ (µg/m^3^) | 1.27 (-6.86, 9.39) | 2.72 (0.329, 5.12) | 3.64 (0.362, 6.93) |
| NO_2_ (ppb) | 1.17 (-6.84, 9.19) | 2.62 (0.293, 4.94) | 3.58 (0.411, 6.74) |
| O_3_ (ppb) | 1.37 (-6.60, 9.33) | 2.71 (0.328, 5.08) | 3.44 (0.241, 6.63) |
| Time between study days | 1.32 (-6.64, 9.28) | 2.56 (0.243, 4.87) | 3.11 (-0.126, 6.36) |
| Regional Air Quality^c^ |  |  |  |
| 24-hour mean PM_2.5_ | 0.925 (-7.39, 9.24) | 2.71 (0.113, 5.30) | 2.73 (-0.779, 6.23) |
| 24-hour mean NO_2_ | 1.19 (-6.79, 9.18) | 2.64 (0.274, 5.00) | 3.34 (0.063, 6.63) |
| 24-hour mean O_3_ | 1.26 (-6.69, 9.21) | 2.51 (0.147, 4.87) | 3.32 (0.129, 6.52) |
| 5-day mean PM_2.5_ | 0.705 (-7.67, 9.08) | 2.46 (-0.118, 5.04) | 2.90 (-0.656, 6.45) |
| 5-day mean NO_2_ | -0.393 (-8.93, 8.14) | 2.20 (-0.344, 4.75) | 2.41 (-1.08, 5.91) |
| 5-day mean O_3_ | 1.39 (-6.67, 9.45) | 2.19 (-0.163, 4.54) | 3.38 (0.0874, 6.68) |

RHI, reactive hyperaemia index; BP, blood pressure; UFP, ultrafine particles. ^a^Model adjusted for ambient temperature during exercise, mean heart rate during exercise, and alcohol/caffeine consumption in the past 24-hours. Regression coefficients reflect interquartile range increases in exposure and are mutually adjusted for all other air pollutants.^b^ Additional adjustment (separately) for exposures during the preceding study period.^c^ Additional adjustment (separately) for regional air quality in the 24-hours and 5-days prior to the study period.

Table S8 (continued). Multi-Pollutant Models for the Relationship between Air Pollution Exposures and Percent Changes in Reactive Hyperemia Index and Blood Pressure 3-hours after Exercise with Additional Adjustment for Exposures during Previous Visits and Regional Air Quality

| Exposure | Percent Change in Outcome | | |
| --- | --- | --- | --- |
|  | RHI  β (95% CI) | Systolic BP  β (95% CI) | Diastolic BP  β (95% CI) |
| Black Carbon^a^ | 0.546 (-2.84, 3.94) | -0.264 (-1.26, 0.734) | -0.233 (-1.61, 1.15) |
| Adjusted (separately) for: | |  |  |
| Previous Exposures^b^: |  |  |  |
| UFPs (count/cm^3^) | 0.339 (-3.10, 3.74) | -0.337 (-1.33, 0.658) | -0.316 (-1.68, 1.05) |
| Black Carbon (ηg/m^3^) | 0.602 (-2.79, 4.00) | -0.158 (-1.14, 0.826) | -0.118 (-1.50, 1.26) |
| PM_2.5_ (µg/m^3^) | 0.587 (-2.86, 4.03) | -0.154 (-1.15, 0.846) | -0.0347 (-1.41, 1.34) |
| NO_2_ (ppb) | 0.588 (-2.84, 4.02) | -0.353 (-1.34, 0.636) | -0.361 (-1.71, 0.989) |
| O_3_ (ppb) | 0.378 (-3.05, 3.80) | -0.393 (-1.38, 0.593) | -0.393 (-1.76, 0.975) |
| Time between study days | 0.569 (-2.84, 3.98) | -0.356 (-1.33, 0.621) | -0.301 (-1.68, 1.08) |
| Regional Air Quality^c^ | |  |  |
| 24-hour mean PM_2.5_ | 1.31 (-2.51, 5.12) | -0.230 (-1.39, 0.926) | -0.496 (-0.237, 4.76) |
| 24-hour mean NO_2_ | 0.580 (-2.85, 4.01) | -0.329 (-1.34, 0.680) | -0.257 (-1.66, 1.14) |
| 24-hour mean O_3_ | 0.558 (-2.85, 3.96) | -0.251 (-1.26, 0.754) | -0.139 (-1.50, 1.22) |
| 5-day mean PM_2.5_ | 0.640 (-3.07, 4.35) | -0.281 (-1.38, 0.824) | -0.458 (-1.98, 1.06) |
| 5-day mean NO_2_ | 0.424 (-2.97, 3.82) | -0.287 (-1.29, 0.719) | -0.326 (-1.70, 1.05) |
| 5-day mean O_3_ | 0.613 (-2.86, 4.09) | -0.456 (-1.47, 0.556) | -0.456 (-1.47, 0.556) |
| PM_2.5_^a^ | 4.11 (-2.09, 10.3) | 0.775 (-1.08, 2.63) | -1.31 (-3.88, 1.25) |
| Adjusted (separately) for: |  |  |  |
| Previous Exposures^b^: |  |  |  |
| UFPs (count/cm^3^) | 3.59 (-2.64, 9.84) | 0.831 (-1.03, 2.69) | -1.13 (-3.67, 1.42) |
| Black Carbon (ηg/m^3^) | 3.57 (-2.70, 9.86 | 1.10 (-0.747, 2.95) | -0.927 (-3.51, 1.66) |
| PM_2.5_ (µg/m^3^) | 4.29 (-2.04, 10.6) | 0.875 (-0.987, 2.74) | -1.16 (-3.72, 1.39) |
| NO_2_ (ppb) | 3.91 (-3.26, 11.1) | 1.17 (-0.916, 3.26) | -0.633 (-3.47, 2.21) |
| O_3_ (ppb) | 4.12 (-2.97, 11.2) | 1.22 (-0.963, 3.41) | -1.04 (-3.89, 1.81) |
| Time between study days | 4.07 (-2.18, 10.3) | 0.958 (-1.11, 3.02) | -1.23 (-3.79, 1.33) |
| Regional Air Quality^c^ |  |  |  |
| 24-hour mean PM_2.5_ | 3.73 (-2.52, 9.98) | 0.707 (-1.24, 2.65) | -1.26 (-3.89, 1.37) |
| 24-hour mean NO_2_ | 4.10 (-2.14, 10.3) | 0.790 (-1.07, 2.65) | -1.30 (-3.87, 1.28) |
| 24-hour mean O_3_ | 4.19 (-2.10, 10.5) | 0.823 (-1.05, 2.70) | -0.965 (-3.50, 1.57) |
| 5-day mean PM_2.5_ | 2.40 (-4.14, 8.93) | 0.227 (-1.76, 2.22) | -1.49 (-4.23, 1.25) |
| 5-day mean NO_2_ | 3.64 (-2.63, 9.90) | 0.708 (-1.17, 2.59) | -1.52 (-4.10, 1.05) |
| 5-day mean O_3_ | 4.30 (-2.23, 10.8) | 0.302 (-1.62, 2.23) | 0.302 (-1.62, 2.23) |

RHI, reactive hyperaemia index; BP, blood pressure; UFP, ultrafine particles. ^a^Model adjusted for ambient temperature during exercise, mean heart rate during exercise, and alcohol/caffeine consumption in the past 24-hours. Regression coefficients reflect interquartile range increases in exposure and are mutually adjusted for all other air pollutants.^b^ Additional adjustment (separately) for exposures during the preceding study period.^c^ Additional adjustment (separately) for regional air quality in the 24-hours and 5-days prior to the study period.

Table S8 (continued). Multi-Pollutant Models for the Relationship between Air Pollution Exposures and Percent Changes in Reactive Hyperemia Index and Blood Pressure 3-hours after Exercise with Additional Adjustment for Exposures during Previous Visits and Regional Air Quality

| Exposure | Percent Change in Outcome | | |
| --- | --- | --- | --- |
|  | RHI  β (95% CI) | Systolic BP  β (95% CI) | Diastolic BP  β (95% CI) |
| NO_2_^a^ | -1.52 (-7.31, 4.28) | -0.559 (-2.26, 1.14) | -1.35 (-3.70, 0.991) |
| Adjusted (separately) for: | |  |  |
| Previous Exposures^b^: |  |  |  |
| UFPs (count/cm^3^) | -0.270 (-6.26, 5.73) | -0.824 (-2.57, 0.926) | -1.96 (-4.36, 0.439) |
| Black Carbon (ηg/m^3^) | -1.18 (-7.07, 4.71) | -1.00 (-2.70, 0.690) | -1.80 (-4.19, 0.590) |
| PM_2.5_ (µg/m^3^) | -1.41 (-7.36, 4.53) | -0.860 (-2.58, 0.865) | -1.84 (-4.21, 0.532) |
| NO_2_ (ppb) | -1.35 (-7.29, 4.58) | -0.868 (-2.56, 0.825) | -1.94 (-4.25, 0.381) |
| O_3_ (ppb) | -2.19 (-8.16, 3.77) | -1.11 (-2.91, 0.685) | -2.06 (-4.44, 0.309) |
| Time between study days | -1.42 (-7.33, 4.48) | -1.04 (-2.75, 0.668) | -1.62 (-3.99, 0.744) |
| Regional Air Quality^c^ | |  |  |
| 24-hour mean PM_2.5_ | -0.843 (-7.00, 5.31) | -0.898 (-2.78, 0.987) | -1.35 (-3.90, 1.20) |
| 24-hour mean NO_2_ | -1.44 (-7.32, 4.45) | -0.691 (-2.41, 1.03) | -1.43 (-3.81, 0.962) |
| 24-hour mean O_3_ | -1.50 (-7.31, 4.32) | -0.547 (-2.25, 1.16) | -1.29 (-3.60, 1.02) |
| 5-day mean PM_2.5_ | -0.214 (-6.55, 6.13) | -0.320 (-2.24, 1.61) | -1.34 (-4.00, 1.32) |
| 5-day mean NO_2_ | -1.87 (-7.70, 3.95) | -0.592 (-2.30, 1.11) | -1.46 (-3.80, 0.877) |
| 5-day mean O_3_ | -1.53 (-7.35, 4.29) | -0.568 (-2.24, 1.11) | -0.568 (-2.24, 1.11) |

RHI, reactive hyperaemia index; BP, blood pressure; UFP, ultrafine particles. ^a^Model adjusted for ambient temperature during exercise, mean heart rate during exercise, and alcohol/caffeine consumption in the past 24-hours. Regression coefficients reflect interquartile range increases in exposure and are mutually adjusted for all other air pollutants.^b^ Additional adjustment (separately) for exposures during the preceding study period.^c^ Additional adjustment (separately) for regional air quality in the 24-hours and 5-days prior to the study period.

Table S9. Multi-Pollutant Models for the Relationship between Personal UFP Exposures and Acute Changes in HRV over the entire follow-up period with additional adjustment for exposures during previous visits and regional air quality

|  | Heart Rate Variability | | | | | |
| --- | --- | --- | --- | --- | --- | --- |
|  | SDNN (ms)  β (95% CI) | RMSSD (ms)  β (95% CI) | pNN50 (%)  β (95% CI) | LF (ms^2^)  β (95% CI) | HF (ms^2^)  β (95% CI) | LF:HF  β (95% CI) |
| UFP^a^ | 3.61 (0.227, 7.00) | 0.920 (-0.442, 2.28) | 0.680 (-0.445, 1.81) | 60.3 (-32.8, 153) | 24.7 (-18.5, 67.9) | 0.0445  (-0.337, 0.426) |
| Adjusted (separately) for: | |  |  |  |  |  |
| Previous Exposures^b^: |  |  |  |  |  |  |
| UFPs (count/cm^3^) | 3.95 (0.412, 7.50) | 1.68 (0.243, 3.12) | 1.24 (0.0572, 2.43) | 55.4 (-41.8, 153) | 32.3 (-13.6, 78.1) | 0.0568  (-0.341, 0.455) |
| Black Carbon (ηg/m^3^) | 4.09 (0.700, 7.48) | 1.20 (-0.177, 2.58) | 0.870 (-0.270, 2.01) | 52.6 (-40.8, 146) | 32.9 (-10.9, 76.6) | 0.0366  (-0.350, 0.423) |
| PM_2.5_ (µg/m^3^) | 3.74 (0.346, 7.14) | 1.01 (-0.353, 2.37) | 0.803 (-0.325, 1.93) | 62.7 (-30.5, 156) | 32.2 (-11.1, 75.5) | 0.0235  (-0.362, 0.409) |
| NO_2_ (ppb) | 4.20 (0.855, 7.55) | 1.32 (-0.0059, 2.64) | 0.980 (-0.127, 2.09) | 55.4 (-37.7, 148) | 36.4 (-6.67, 79.5) | 0.0155  (-0.367, 0.398) |
| O_3_ (ppb) | 4.05 (0.721, 7.38) | 1.18 (-0.149, 2.52) | 0.883 (-0.228, 1.99) | 54.6 (-38.3, 147) | 34.8 (-8.19, 77.9) | 0.00740  (-0.374, 0.389) |
| Time between study days | 3.60 (0.204, 6.99) | 0.983 (-0.380, 2.35) | 0.714 (-0.415, 1.84) | 54.9 (-37.6, 147) | 30.2 (-12.9, 73.3) | 0.0470  (-0.336, 0.429) |
| Regional Air Quality^c^ |  |  |  |  |  |  |
| 24-hour mean PM_2.5_ | 5.90 (0.913, 10.9) | 1.72 (-0.268, 3.71) | 0.951 (-0.675, 2.58) | 95.4 (-37.5, 228) | 14.4 (-47.3, 76.2) | -0.233  (-0.782, 0.316) |
| 24-hour mean NO_2_ | 3.19 (-0.160, 6.55) | 0.826 (-0.534, 2.19) | 0.623 (-0.504, 1.75) | 48.9 (-43.3, 141) | 28.6 (-14.5, 71.7) | 0.0489  (-0.334, 0.432) |
| 24-hour mean O_3_ | 2.25 (-1.28, 5.77) | 1.02 (-0.403, 2.44) | 0.800 (-0.374, 1.97) | 49.6 (-46.5, 146) | 29.6 (-15.4, 74.5) | 0.0229  (-0.375, 0.421) |
| 5-day mean PM_2.5_ | 5.20 (0.313, 10.1) | 1.56 (-0.401, 3.51) | 0.769 (-0.846, 2.38) | 83.3 (-50.7, 217) | 25.2 (-36.2, 86.6) | -0.193  (-0.739, 0.352) |
| 5-day mean NO_2_ | 3.27 (-0.308, 6.85) | 0.952 (-0.492, 2.40) | 0.620 (-0.575, 1.82) | 52.0 (-46.0, 150) | 26.9 (-18.8, 72.5) | 0.0241  (-0.379,0.427) |
| 5-day mean O_3_ | 2.23 (-1.30, 5.76) | 1.13 (-0.292, 2.55) | 0.914 (-0.259, 2.09) | 81.2 (-14.5, 177) | 38.2 (-6.62, 83.0) | 0.0376  (-0.362, 0.437) |

^a^Model adjusted for ambient temperature during exercise, mean heart rate during exercise, and alcohol/caffeine consumption in the past 24-hours;^b^Additional adjustment (separately) for exposures during the preceding study period; ^c^Additional adjustment (separately) for regional air quality in the 24-hours and 5-days prior to the study period. Regression coefficients reflect interquartile range increases in exposure and are mutually adjusted for all other air pollutants.

Table S10. Multi-Pollutant Models for the Relationship between Personal Black Carbon Exposures and Acute Changes in HRV over the entire follow-up period with additional adjustment for exposures during previous visits and regional air quality

|  | Heart Rate Variability | | | | | |
| --- | --- | --- | --- | --- | --- | --- |
|  | SDNN (ms)  β (95% CI) | RMSSD (ms)  β (95% CI) | pNN50 (%)  β (95% CI) | LF (ms^2^)  β (95% CI) | HF (ms^2^)  β (95% CI) | LF:HF  β (95% CI) |
| Black Carbon^a^ | 3.56 (0.865, 6.25) | -0.460 (-1.53, 0.615) | -0.483 (-1.37, 0.403) | 66.3 (-6.48, 139) | 9.52  (-24.5, 43.6) | 0.0919  (-0.209, 0.392) |
| Adjusted (separately) for: | |  |  |  |  |  |
| Previous Exposures^b^: |  |  |  |  |  |  |
| UFPs (count/cm^3^) | 4.43 (1.78, 7.09) | -0.430 (-1.51, 0.648) | -0.485 (-1.38, 0.406) | 68.2 (-5.02, 141) | 5.68  (-28.8, 40.1) | 0.0163  (-0.281, 0.314) |
| Black Carbon (ηg/m^3^) | 4.11 (1.34, 6.88) | -0.118 (-1.22, 0.982) | -0.265 (-1.17, 0.640) | 69.0 (-4.74, 143) | 10.1  (-24.8, 45.0) | 0.0678  (-0.241, 0.377) |
| PM_2.5_ (µg/m^3^) | 3.90 (1.19, 6.60) | -0.363 (-1.44, 0.713) | -0.377 (-1.27, 0.511) | 64.6 (-8.57, 138) | 7.23  (-26.9, 41.4) | 0.124  (-0.178, 0.427) |
| NO_2_ (ppb) | 3.65 (0.997, 6.31) | -0.500 (-1.53, 0.543) | -0.520 (-1.39, 0.348) | -176 (-297, -55.9) | 4.61  (-29.3, 38.5) | 0.102  (-0.198, 0.403) |
| O_3_ (ppb) | 3.59 (0.937, 6.24) | -0.550 (-1.60, 0.503) | -0.558 (-1.43, 0.318) | 66.7 (-6.26, 140) | 3.23  (-30.7, 37.2) | 0.119  (-0.181, 0.420) |
| Time between study days | 3.57 (0.870, 6.27) | -0.506 (-1.58, 0.569) | -0.508 (-1.40, 0.381) | 63.9 (-8.69, 136) | 4.89  (-29.1, 38.9) | 0.900  (-0.211, 0.391) |
| Regional Air Quality^c^ |  |  |  |  |  |  |
| 24-hour mean PM_2.5_ | 2.14 (-0.854, 5.13) | -0.843 (-2.06, 0.371) | -0.646 (-1.64, 0.348) | 40.0 (-41.5, 122) | 12.3 (-25.3, 49.9) | 0.180  (-0.153, 0.514) |
| 24-hour mean NO_2_ | 2.94 (0.250, 5.62) | -0.610 (-1.69, 0.472) | -0.584 (-1.48, 0.310) | 59.4 (-13.6, 132) | 4.12 (-30.2, 38.4) | 0.0995  (-0.204, 0.403) |
| 24-hour mean O_3_ | 3.23 (0.538, 5.92) | -0.441 (-1.52, 0.636) | -0.464 (-1.35, 0.425) | 65.1 (-7.42, 138) | 5.47 (-28.5, 39.5) | 0.0874  (-0.214,0.389) |
| 5-day mean PM_2.5_ | 3.12 (0.261, 5.98) | -0.551 (-1.72, 0.612) | -0.430 (-1.39, 0.536) | 56.1 (-24.4, 137) | 8.17 (-28.3, 44.7) | 0.160  (-0.163, 0.482) |
| 5-day mean NO_2_ | 3.65 (0.941, 6.36) | -0.467 (-1.55, 0.614) | -0.470 (-1.36, 0.421) | 65.5 (-7.22, 138) | 6.00 (-28.1, 40.1) | 0.0967  (-0.206, 0.399) |
| 5-day mean O_3_ | 2.59 (-0.180, 5.37) | -0.321 (-1.43, 0.786) | -0.333 (-1.24, 0.578) | 83.8 (9.59, 158) | 11.1 (-23.8, 46.0) | 0.0872  (-0.223, 0.397) |

^a^Model adjusted for ambient temperature during exercise, mean heart rate during exercise, and alcohol/caffeine consumption in the past 24-hours;^b^Additional adjustment (separately) for exposures during the preceding study period; ^c^Additional adjustment (separately) for regional air quality in the 24-hours and 5-days prior to the study period. Regression coefficients reflect interquartile range increases in exposure and are mutually adjusted for all other air pollutants.

Table S11. Multi-Pollutant Models for the Relationship between Personal PM_2.5_ Exposures and Acute Changes in HRV over the entire follow-up period with additional adjustment for exposures during previous visits and regional air quality

|  | Heart Rate Variability | | | | | |
| --- | --- | --- | --- | --- | --- | --- |
|  | SDNN (ms)  β (95% CI) | RMSSD (ms)  β (95% CI) | pNN50 (%)  β (95% CI) | LF (ms^2^)  β (95% CI) | HF (ms^2^)  β (95% CI) | LF:HF  β (95% CI) |
| PM_2.5_^a^ | -1.24 (-6.32, 3.84) | -2.73 (-4.73, -0.721) | -2.31 (-3.96, -0.665) | 37.3 (-97.6, 172) | -44.0  (-107, 19.6) | -0.00579  (-0.570, 0.559) |
| Adjusted (separately) for: | |  |  |  |  |  |
| Previous Exposures^b^: |  |  |  |  |  |  |
| UFPs (count/cm^3^) | -0.738 (-5.74, 4.27) | -2.37 (-4.38, -0.350) | -2.08 (-3.74, -0.418) | 41.6 (-94.1, 177) | -43.6  (-108, 20.7) | -0.0746  (-0.633, 0.483) |
| Black Carbon (ηg/m^3^) | 0.129 (-4.99, 5.24) | -2.41 (-4.45, -0.382) | -2.10 (-3.78, -0.429) | 46.1 (-90.0, 183) | -39.6  (-104, 24.9) | -0.0002004  (-0.573, 0.572) |
| PM_2.5_ (µg/m^3^) | -0.644 (-5.78, 4.50) | -2.75 (-4.77, -0.729) | -2.25 (-3.91, -0.586) | 26.9 (-109, 163) | -44.5  (-108, 19.6) | 0.0785  (-0.491, 0.647) |
| NO_2_ (ppb) | -4.42 (-10.0, 1.18) | -3.59 (-5.78, -1.40) | -2.94 (-4.76, -1.13) | -7.25 (-159, 144) | -57.2  (-128, 13.8) | -0.0495  (-0.681, 0.582) |
| O_3_ (ppb) | -5.19 (-10.7, 0.353) | -4.17 (-6.36, -1.99) | -3.36 (-5.17, -1.54) | -10.7 (-161, 140) | -66.6  (-137, 3.78) | -0.00801  (-0.633, 0.617) |
| Time between study days | -1.23 (-6.32, 3.86) | -2.75 (-4.75, -0.740) | -2.32 (-3.98, -0.673) | 36.2 (-98.1, 170) | -45.5  (-109, 17.8) | -0.00652  (-0.572, 0.559) |
| Regional Air Quality^c^ |  |  |  |  |  |  |
| 24-hour mean PM_2.5_ | -0.669 (-5.82, 4.47) | -2.66 (-4.72, -0.605) | -2.35 (-4.03, -0.667) | 42.5 (-94.6, 180) | -53.8  (-118, 9.91) | -0.0364  (-0.603, 0.531) |
| 24-hour mean NO_2_ | -2.05 (-7.10, 2.99) | -2.90 (-4.91, -0.890) | -2.42 (-4.08, -0.771) | 30.9 (-103, 165) | -46.7  (-110, 16.7) | 0.00241  (-0.565, 0.569) |
| 24-hour mean O_3_ | -1.56 (-6.62, 3.51) | -2.69 (-4.71, -0.677) | -2.27 (-3.92, -0.610) | 34.5 (-100, 169) | -45.4  (-109, 18.1) | -0.0142  (-0.581, 0.552) |
| 5-day mean PM_2.5_ | 0.661 (-4.66, 5.98) | -2.23 (-4.36, -0.0934) | -1.96 (-3.72, -0.197) | 79.9 (-66.1, 226) | -53.0  (-120, 13.8) | -0.0807  (-0.675, 0.513) |
| 5-day mean NO_2_ | -1.11 (-6.22, 4.00) | -2.74 (-4.76, -0.721) | -2.29 (-3.95, -0.630) | 35.9 (-99.3, 171) | -44.4  (-108, 19.3) | 0.00403  (-0.564, 0.573) |
| 5-day mean O_3_ | -2.90 (-8.12, 2.32) | -2.47 (-4.54, -0.395) | -2.03 (-3.73, -0.323) | 71.6 (-66.9, 210) | -34.2  (-100, 31.1) | -0.0148  (-0.599, 0.569) |

^a^Model adjusted for ambient temperature during exercise, mean heart rate during exercise, and alcohol/caffeine consumption in the past 24-hours;^b^Additional adjustment (separately) for exposures during the preceding study period; ^c^Additional adjustment (separately) for regional air quality in the 24-hours and 5-days prior to the study period. Regression coefficients reflect interquartile range increases in exposure and are mutually adjusted for all other air pollutants.

Table S12. Multi-Pollutant Models for the Relationship between Personal NO_2_ Exposures and Acute Changes in HRV over the entire follow-up period with additional adjustment for exposures during previous visits and regional air quality

|  | Heart Rate Variability | | | | | |
| --- | --- | --- | --- | --- | --- | --- |
|  | SDNN (ms)  β (95% CI) | RMSSD (ms)  β (95% CI) | pNN50 (%)  β (95% CI) | LF (ms^2^)  β (95% CI) | HF (ms^2^)  β (95% CI) | LF:HF  β (95% CI) |
| NO_2_^a^ | -5.92 (-10.3, -1.50) | 0.347 (-1.41, 2.10) | 0.553 (-0.89, 2.00) | -187 (-306, -67.8) | -8.85 (-64.5, 46.8) | -0.401  (-0.895, 0.0935) |
| Adjusted (separately) for: | |  |  |  |  |  |
| Previous Exposures^b^: |  |  |  |  |  |  |
| UFPs (count/cm^3^) | -7.76 (-12.3, -3.21) | -0.556 (-2.39, 1.28) | -0.0784 (-1.59, 1.44) | -181 (-305, -56.5) | -18.1 (-76.9, 40.7) | -0.246  (-0.755, 0.262) |
| Black Carbon (ηg/m^3^) | -7.14 (-11.7, -2.63) | -0.161 (-1.96, 1.64) | 0.208 (-1.28, 1.70) | -186 (-307, -64) | -21.8 (-79.2, 35.6) | -0.386  (-0.894, 1.22) |
| PM_2.5_ (µg/m^3^) | -6.51 (-11.1, -1.94) | 0.310 (-1.49, 2.11) | 0.389 (-1.10, 1.88) | -159 (-281, -37.1) | -15.1 (-72.4, 42.2) | -0.532  (-1.04, -0.0241) |
| NO_2_ (ppb) | -6.48 (-10.9, -2.09) | -0.161 (-1.88, 1.56) | 0.167 (-1.27, 1.60) | -176 (-297, -55.9) | -23.0 (-79.1, 33.1) | -0.353  (-0.851, 0.145) |
| O_3_ (ppb) | -6.93 (-11.4, -2.46) | -0.413 (-2.19, 1.36) | 0.0101 (-1.46, 1.48) | -177 (-300, -54.3) | -28.9 (-86.2, 28.3) | -0.261  (-0.768, 0.247) |
| Time between study days | -5.88 (-10.3, -1.43) | 0.195 (-1.58, 1.97) | 0.477 (-0.985, 1.94) | -181 (-300, -60.9) | -15.7 (-71.8, 40.4) | -0.407  (-0.906, 0.0908) |
| Regional Air Quality^c^ |  |  |  |  |  |  |
| 24-hour mean PM_2.5_ | -4.49 (-9.30, 0.324) | 0.464 (-1.46, 2.38) | 0.355 (-1.21, 1.92) | -163 (-291, -34.5) | -17.0 (-76.6, 42.6) | -0.533  (-1.06, -0.00414) |
| 24-hour mean NO_2_ | -7.05 (-11.5, -2.63) | 0.104 (-1.67, 1.88) | 0.393 (-1.07, 1.86) | -185 (-304, -65.2) | -16.1 (-72.3, 40.1) | -0.388  (-0.888, 0.112) |
| 24-hour mean O_3_ | -6.13 (-10.5, -1.72) | 0.366 (-1.40, 2.13) | 0.576 (-0.875, 2.03) | -175 (-294, -56.5) | -13.8 (-69.5, 41.8) | -0.408  (-0.903, 0.0876) |
| 5-day mean PM_2.5_ | -4.48 (-9.61, 0.640) | 0.543 (-1.51, 2.59) | 0.333 (-1.36, 2.02) | -166 (-306, -25.9) | -8.93 (-73.3, 55.4) | -0.568  (-1.14, 0.00212) |
| 5-day mean NO_2_ | -5.84 (-10.3, -1.41) | 0.337 (-1.43, 2.10) | 0.571 (-0.884, 2.03) | -174 (-293, -55.7) | -13.2 (-69.0, 42.6) | -0.396  (-0.892, 0.100) |
| 5-day mean O_3_ | -5.95 (-10.3, -1.54) | 0.365 (-1.39, 2.12) | 0.575 (-0.873, 2.02) | -173 (-291, -54.6) | -13.2 (-68.8, 42.4) | -0.402  (-0.897, 0.0934) |

^a^Model adjusted for ambient temperature during exercise, mean heart rate during exercise, and alcohol/caffeine consumption in the past 24-hours;^b^Additional adjustment (separately) for exposures during the preceding study period; ^c^Additional adjustment (separately) for regional air quality in the 24-hours and 5-days prior to the study period. Regression coefficients reflect interquartile range increases in exposure and are mutually adjusted for all other air pollutants.

Table S13. Multi-Pollutant Models for the Relationship between Personal O_3_ Exposures and Acute Changes in HRV over the entire follow-up period with additional adjustment for exposures during previous visits and regional air quality

|  | Heart Rate Variability | | | | | |
| --- | --- | --- | --- | --- | --- | --- |
|  | SDNN (ms)  β (95% CI) | RMSSD (ms)  β (95% CI) | pNN50 (%)  β (95% CI) | LF (ms^2^)  β (95% CI) | HF (ms^2^)  β (95% CI) | LF:HF  β (95% CI) |
| O_3_^a^ | 14.9 (8.32, 21.6) | 2.23 (-0.325, 4.78) | 1.47 (-0.623, 3.56) | 271 (100, 441) | -0.992 (-81.5, 79.6) | 0.200 (-0.535, 0.936) |
| Adjusted (separately) for: | |  |  |  |  |  |
| Previous Exposures^b^: |  |  |  |  |  |  |
| UFPs (count/cm^3^) | 16.9 (10.1, 23.7) | 2.77 (0.122, 5.41) | 1.81 (-0.348, 3.98) | 273 (98.9, 447) | -2.02 (-85.8, 81.8) | 0.0906  (-0.658, 0.839) |
| Black Carbon (ηg/m^3^) | 15.5 (8.88, 22.1) | 2.14 (-0.438, 4.72) | 1.39 (-0.716, 3.50) | 267 (96.5, 437) | -1.64 (-83.1, 79.8) | 0.206 (-0.535, 0.947) |
| PM_2.5_ (µg/m^3^) | 13.8 (6.70, 20.9) | 1.52 (-1.17, 4.22) | 1.31 (-0.888, 3.50) | 235 (57.6, 413) | -9.00 (-93.9, 75.9) | 0.437 (-0.333, 1.21) |
| NO_2_ (ppb) | 15.2 (8.67, 21.7) | 2.16 (-0.328, 4.66) | 1.41 (-0.647, 3.46) | 250 (80.7, 419) | -4.11 (-84.4, 76.2) | 0.223 (-0.512, 0.959) |
| O_3_ (ppb) | 15.3 (8.83, 21.9) | 2.15 (-0.361, 4.66) | 1.37 (-0.696, 3.44) | 249 (79.8, 419) | -4.83 (-85.2, 75.5) | 0.218 (-0.515, 0.951) |
| Time between study days | 15.0 (8.33, 21.7) | 2.00 (-0.581, 4.57) | 1.35 (-0.754, 3.46) | 247 (77.4, 417) | -4.97 (-86.0, 76.0) | 0.189 (-0.553,0.932) |
| Regional Air Quality^c^ |  |  |  |  |  |  |
| 24-hour mean PM_2.5_ | 13.5 (6.41, 20.6) | 1.85 (-0.924, 4.62) | 1.46 (-0.789, 3.72) | 227 (45.2, 410) | 2.15 (-83.5, 87.8) | 0.333 (-0.438, 1.10) |
| 24-hour mean NO_2_ | 16.4 (9.81, 23.0) | 2.52 (-0.044, 5.09) | 1.66 (-0.448, 3.76) | 265 (95.5, 435) | 0.234 (-80.7, 81.2) | 0.186 (-0.554, 0.926) |
| 24-hour mean O_3_ | 14.3 (7.70, 20.8) | 2.25 (-0.309, 4.81) | 1.49 (-0.604, 3.58) | 253 (84.5, 422) | -2.25 (-82.7, 78.2) | 0.198 (-0.538, 0.935) |
| 5-day mean PM_2.5_ | 13.8 (6.65, 21.0) | 1.88 (-0.932, 4.69) | 1.48 (-0.825, 3.79) | 229 (39.9, 418) | -3.95 (-91.7, 83.8) | 0.375 (-0.413, 1.16) |
| 5-day mean NO_2_ | 15.7 (8.63, 22.8) | 2.17 (-0.570, 4.90) | 1.59 (-0.652, 3.82) | 254 (73.8, 435) | 2.64 (-83.3, 88.5) | 0.245 (-0.544, 1.03) |
| 5-day mean O_3_ | 13.5 (6.83, 20.1) | 2.40 (-0.175, 4.98) | 1.65 (-0.456, 3.76) | 278 (108, 448) | 4.71 (-76.3, 85.7) | 0.194 (-0.549, 0.937) |

^a^Model adjusted for ambient temperature during exercise, mean heart rate during exercise, and alcohol/caffeine consumption in the past 24-hours;^b^Additional adjustment (separately) for exposures during the preceding study period; ^c^Additional adjustment (separately) for regional air quality in the 24-hours and 5-days prior to the study period. Regression coefficients reflect interquartile range increases in exposure and are mutually adjusted for all other air pollutants.

Table S14. Personal Exposure to PM_2.5_, UFPs, and Black Carbon and Effect Modification by Regional Air Pollution in HRV models

| Outcome | Personal PM_2.5_ | | | | | Personal UFPs | | | | Personal BC | |
| --- | --- | --- | --- | --- | --- | --- | --- | --- | --- | --- | --- |
|  | Low  24h-PM_2.5_ | High  24h-PM_2.5_ | Low  5d-PM_2.5_ | High  5d-PM_2.5_ | | Low  24h-PM_2.5_ | High  24h-PM_2.5_ | Low  24h NO2 | High  24h NO2 | Low  5d-O_3_ | High  5d-O_3_ |
| RMSSD (ms) | 1.10  (-2.40, 4.61) | -7.76  (-10.9, -4.58) | 1.06  (-2.32, 4.44) | -7.30  (-10.5, -4.14) | | - | - | - | - | - | - |
|  | p <0.001^a^ | | p <0.001 ^a^ | | |  |  |  |  |  |  |
| pNN50 | 0.360  (-2.42, 3.14) | -5.76  (-8.40, -3.12) | 0.103  (-2.70, 2.90) | -5.56  (-8.13, -2.98) | | - | - | - | - | - | - |
|  | p <0.001 ^a^ | | p<0.001 ^a^ | | |  |  |  |  |  |  |
| HF (ms^2^) | 6.94  (-103, 117) | -134  (-234, -33.0) | 40.3  (-58.7, 139) | -99.3  (-204, 5.30) | | - | - | - | - | - | - |
|  | p=0.028 ^a^ | | p=0.002 ^a^ | | |  |  |  |  |  |  |
| SDNN  (ms) |  |  |  | |  | -5.65  (-20.1, 8.86) | 0.675  (-4.36, 5.71) | 22.5  (13.1, 31.8) | 0.0323  (-4.89, 4.95) | 5.84  (1.44, 10.2) | -4.56  (-11.8, 2.64) |
|  |  | |  | | |  |  |  |  |  |  |
|  |  |  |  |  |  | p=0.026 ^a^ | | p= 0.015 ^a^ | | p=0.008 ^a^ | |

All models are adjusted for ambient temperature during exercise, mean heart rate during exercise, and alcohol/caffeine consumption in the past 24-hours. High and low cut-off points for stratified analyses are based on median values for regional pollutants: 24h PM_2.5_: 11.5 µg/m^3^; 5d PM_2.5_ median: 13.5 µg/m^3^; 24h NO_2_ median: 6.41 ppb; 5d NO_2_ median: 6.59; 24h O_3_ median: 26.4 ppb; 5d O_3_ median: 26.47 ppb. ^a^ p-value for the first order interaction term between continuous measures of personal air pollutants and regional concentrations.

Table S15. Personal Exposure to O_3_ and Effect Modification by Regional Air Pollution in HRV models

| Outcome | Personal O_3_ | | | | | | | | | |
| --- | --- | --- | --- | --- | --- | --- | --- | --- | --- | --- |
|  | Low  24h-PM_2.5_ | High  24h-PM_2.5_ | Low  5d-PM_2.5_ | High  5d-PM_2.5_ | Low  24h-O_3_ | High  24h-O_3_ | Low  5d-O_3_ | High  5d-O_3_ | Low  5-d NO2 | High  5d NO2 |
| SDNN (ms) | -28.7  (-44.1, -13.2) | 24.0  (15.7, 32.3) | -13.3  (-27.6, -0.979) | 24.3  (16.0, 32.6) | 12.1  (1.22, 22.9) | 12.9  (2.98, 22.8) | 6.81  (-6.25, 19.9) | 16.6  (5.69, 27.4) | -14.0  (-25.8, -2.14) | 26.5  (14.5, 38.5) |
|  | p<0.001 ^a^ | | p<0.001 ^a^ | | p<0.001 ^a^ | | p<0.001 ^a^ | | p<0.001 ^a^ | |
| LF (ms^2^) | -42.5  (-374, 289) | 323  (109, 538) | 235  (-97.3, 567) | 348  (133, 563) | 161  (-78.5, 401) | 182  (-117, 480) | 24.8  (-261, 311) | 412  (117, 708) | 62.6  (-272, 397) | 239  (-32.3, 512) |
|  | p=0.060 ^a^ | | p=0.568 ^a^ | | p=0.838 ^a^ | | p=0.911 ^a^ | | p=0.075 ^a^ | |

All models are adjusted for ambient temperature during exercise, mean heart rate during exercise, and alcohol/caffeine consumption in the past 24-hours. High and low cut-off points for stratified analyses are based on median values for regional pollutants: 24h PM_2.5_: 11.5 µg/m^3^; 5d PM_2.5_ median: 13.5 µg/m^3^; 24h NO_2_ median: 6.41 ppb; 5d NO_2_ median: 6.59; 24h O_3_ median: 26.4 ppb; 5d O_3_ median: 26.47 ppb. ^a^ p-value for the first order interaction term between continuous measures of personal air pollutants and regional concentrations.

Table S16. Personal Exposure to NO_2_ and Effect Modification by Regional Air Pollution in HRV models

| Outcome | NO_2_ | | | | | |
| --- | --- | --- | --- | --- | --- | --- |
|  | Low  24h-PM_2.5_ | High  24h-PM_2.5_ | Low  5d-PM_2.5_ | High  5d-PM_2.5_ | Low  5d-O_3_ | High  5d-O_3_ |
| SDNN (ms) | 7.84 (-0.887, 16.6) | -2.15 (-10.4, 6.06) | 1.40 (-7.06, 9.85) | 6.33 (-1.26, 13.9) | -14.4 (-22.1, -6.68) | -1.44 (-9.75, 6.87) |
|  | p=0.034 ^a^ | | p=0.002 ^a^ | | p=0.001 ^a^ | |

All models are adjusted for ambient temperature during exercise, mean heart rate during exercise, and alcohol/caffeine consumption in the past 24-hours. High and low cut-off points for stratified analyses are based on median values for regional pollutants: 24h PM_2.5_: 11.5 µg/m^3^; 5d PM_2.5_ median: 13.5 µg/m^3^; 24h NO_2_ median: 6.41 ppb; 5d NO_2_ median: 6.59; 24h O_3_ median: 26.4 ppb; 5d O_3_ median: 26.47 ppb. ^a^ p-value for the first order interaction term between continuous measures of personal air pollutants and regional concentrations.
