# Supplementary material for: Exposure to traffic-related air pollution during physical activity and acute changes in blood pressure, autonomic and micro-vascular function in women: a cross-over study
Source: Part Fibre Toxicol. 2014 Dec 9;11:70. doi: 10.1186/s12989-014-0070-4 (PMC4276095; doi:10.1186/s12989-014-0070-4)

ADDITIONAL FILE 2

Exposure to Traffic-Related Air Pollution during Physical Activity and Acute changes in Blood Pressure, Autonomic and Micro-Vascular Function in Women: a Cross-Over Study

Scott Weichenthal*^1^* *Corresponding author Email: [scott.weichenthal@hc-sc.gc.ca](mailto:scott.weichenthal@hc-sc.gc.ca)

Marianne Hatzopoulou^2^ Email: marianne.hatzopoulou@mcgill.ca

Mark S. Goldberg^3^ Email: [mark.goldberg@mcgill.ca](mailto:mark.goldberg@mcgill.ca)

^1^ Air Health Science Division, Health Canada, 269 Laurier Avenue West, K1A 0K9, Ottawa, ON, Canada.

^2^ Department of Civil Engineering, McGill University, Macdonald Engineering Building
817 Sherbrooke Street West, H3A 0C3, Montreal, Quebec, Canada.

^3^ Division of Clinical Epidemiology, McGill University Health Center, 687 Pine Avenue West, H3A 1A1, Montreal, Quebec, Canada.

Figure S1. **Relationship between personal air pollution exposures and hourly changes in time-domain measures of HRV.** All models are adjusted for ambient temperature during exercise, mean heart rate during exercise, and alcohol/caffeine consumption in the past 24-hours. Regression coefficients reflect interquartile range increases in exposure and are mutually adjusted for all other air pollutants.


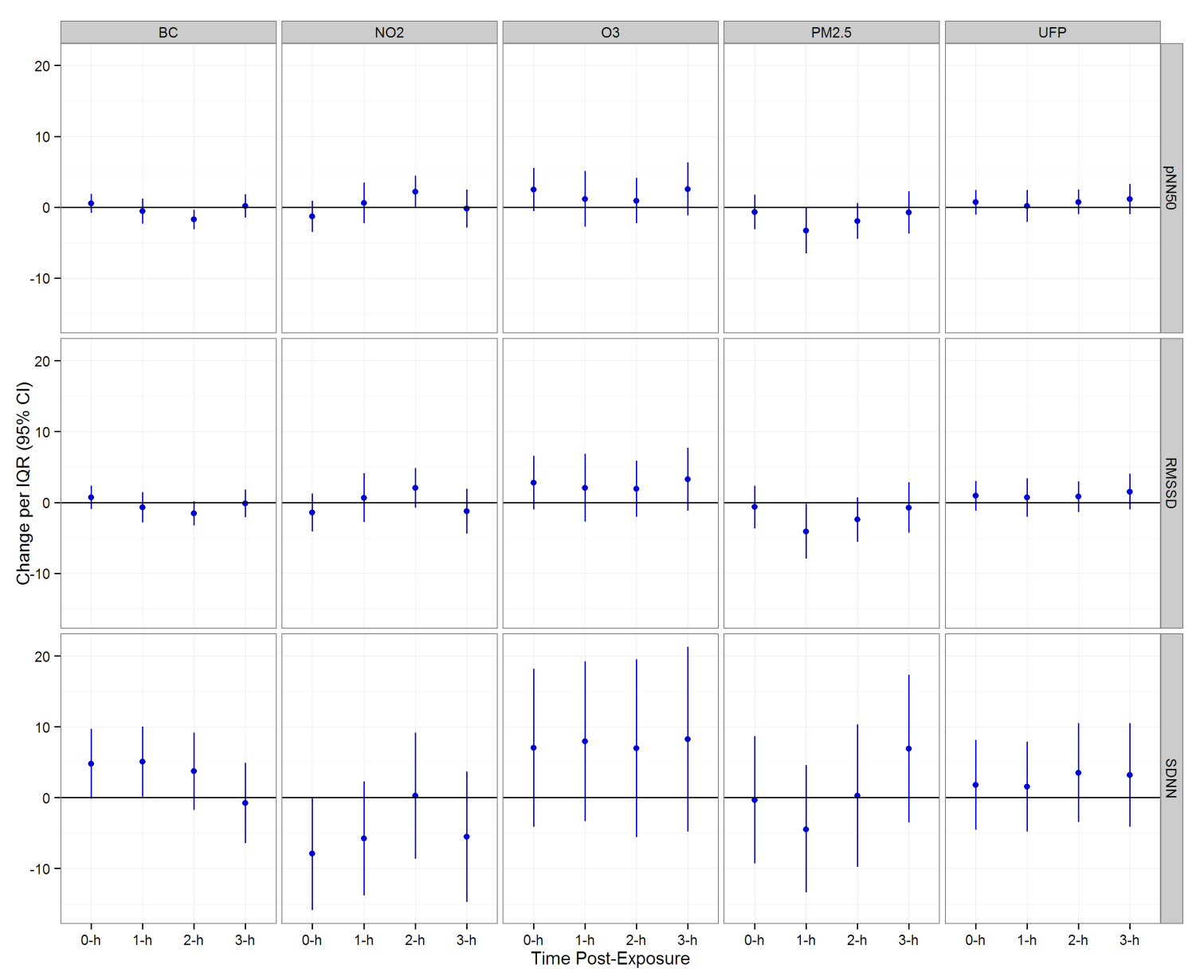


Figure S2. **Relationship between personal air pollution exposures and hourly changes in frequency-domain measures of HRV.** All models are adjusted for ambient temperature during exercise, mean heart rate during exercise, and alcohol/caffeine consumption in the past 24-hours. Regression coefficients reflect interquartile range increases in exposure and are mutually adjusted for all other air pollutants.


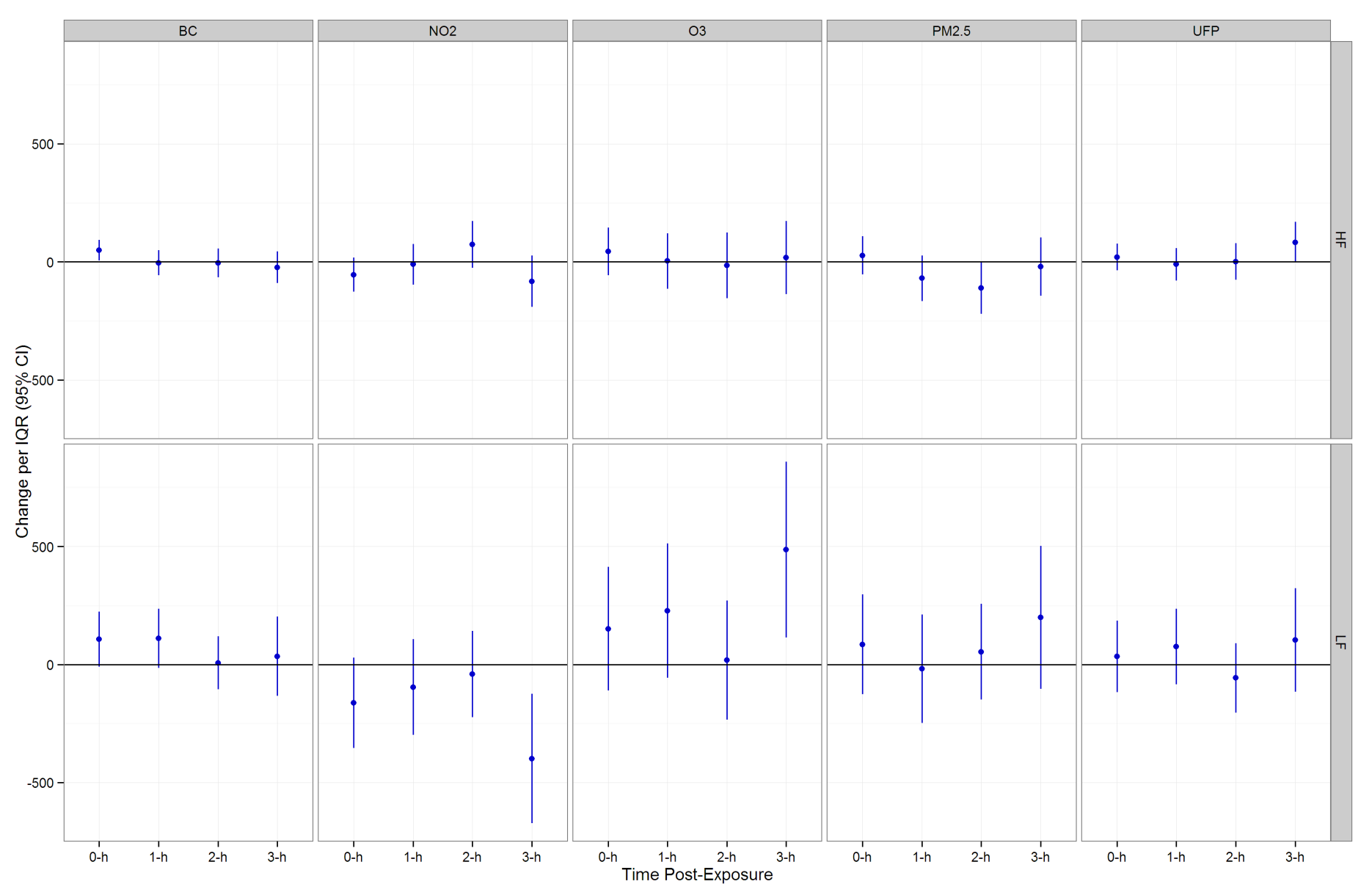


Figure S3: **Map of Cycling Routes**


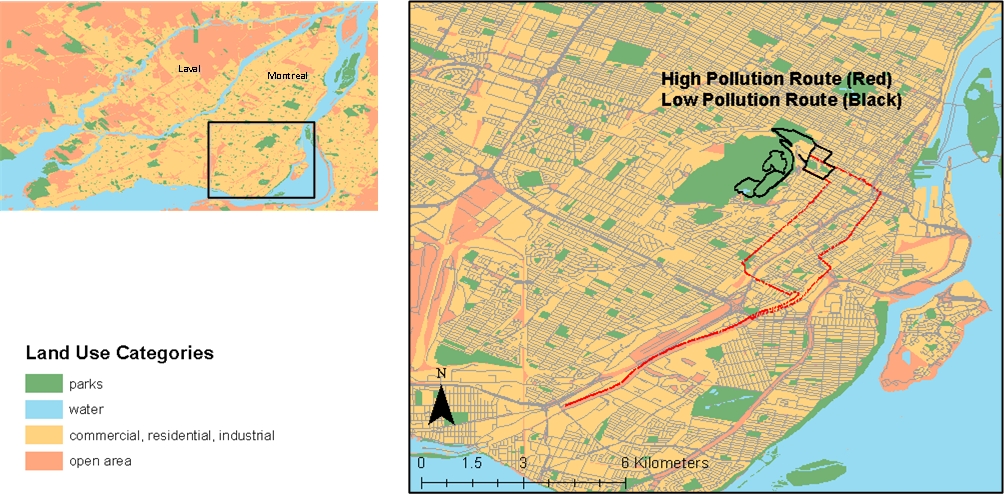

Supplement: Additional file 2: Figure S1. — Relationship between personal air pollution exposures and hourly changes in time-domain measures of HRV. Figure S2: Relationship between personal air pollution exposures and hourly changes in frequency-domain measures of HRV. Figure S3: Map of Cycling Routes. [file 12989_2014_70_MOESM2_ESM.docx]
